# SeqGL Identifies Context-Dependent Binding Signals in Genome-Wide Regulatory Element Maps

**DOI:** 10.1371/journal.pcbi.1004271

**Published:** 2015-05-27

**Authors:** Manu Setty, Christina S. Leslie

**Affiliations:** Computational Biology Program, Memorial Sloan-Kettering Cancer Center, New York, New York, United States of America; University of Massachusetts Medical School, UNITED STATES

## Abstract

Genome-wide maps of transcription factor (TF) occupancy and regions of open chromatin implicitly contain DNA sequence signals for multiple factors. We present SeqGL, a novel *de novo* motif discovery algorithm to identify multiple TF sequence signals from ChIP-, DNase-, and ATAC-seq profiles. SeqGL trains a discriminative model using a *k*-mer feature representation together with group lasso regularization to extract a collection of sequence signals that distinguish peak sequences from flanking regions. Benchmarked on over 100 ChIP-seq experiments, SeqGL outperformed traditional motif discovery tools in discriminative accuracy. Furthermore, SeqGL can be naturally used with multitask learning to identify genomic and cell-type context determinants of TF binding. SeqGL successfully scales to the large multiplicity of sequence signals in DNase- or ATAC-seq maps. In particular, SeqGL was able to identify a number of ChIP-seq validated sequence signals that were not found by traditional motif discovery algorithms. Thus compared to widely used motif discovery algorithms, SeqGL demonstrates both greater discriminative accuracy and higher sensitivity for detecting the DNA sequence signals underlying regulatory element maps. SeqGL is available at http://cbio.mskcc.org/public/Leslie/SeqGL/.

## Author Summary

Transcriptional regulation is the cell’s primary mode of controlling gene expression. Transcription factors (TFs) are proteins that recognize and bind specific DNA sequence signals to regulate the expression of target genes. Recent years have seen the rapid development of genome-wide assays to profile the binding locations of a single TF or, more generally, regions of open chromatin that are occupied by a complex repertoire of DNA binding factors. New methods are therefore needed to detect and represent DNA sequence signals in these genome-wide regulatory element maps. Here we present a novel tool called SeqGL to extract multiple TF binding signals from genome-wide maps. SeqGL employs a machine learning framework to identify features that best discriminate the peaks, where we expect DNA sequence signals to occur, from the flank regions that should not contain these signals. Our tool performed significantly better than widely used motif discovery methods in discriminative accuracy and achieved higher sensitivity in detecting the numerous sequence signals underlying regulatory element maps.

## Introduction

Transcription factor (TF) ChIP-seq profiles and genome-wide regulatory element maps based on DNase I hypersensitive site sequencing (DNase-seq) or transposase-accessible chromatin sequencing (ATAC-seq) implicitly contain rich information about the cell-type specific and genomic-context dependent binding of multiple factors. Traditional analysis of ChIP-seq profiles involves searching for motifs that are significantly enriched in peaks relative to a background model, either using a library of known motifs [1–3] or through de novo motif discovery algorithms [4–9]. However, we hypothesize that motif discovery approaches may miss more subtle cofactor signals that explain a subset of the ChIP peaks and may fail to adequately generalize to the high multiplicity of TF binding signals in DNase profiles. Meanwhile, several methods use DNase-seq profiles to scan for instances of known motifs [10, 11], and one recently proposed approach exploits the read-level properties of high-depth digital genomic footprinting (DGF) to improve localization of known motifs [12]. However, these methods do not enable *de novo* discovery of binding signals that are not represented in TF motif databases, and methods that rely on the depth and read-level properties of DNase I cleavage in DGF may not readily generalize to newer assays like ATAC-seq, which can be used in low cell number settings where DNase-seq is not feasible.

**Fig 1:**
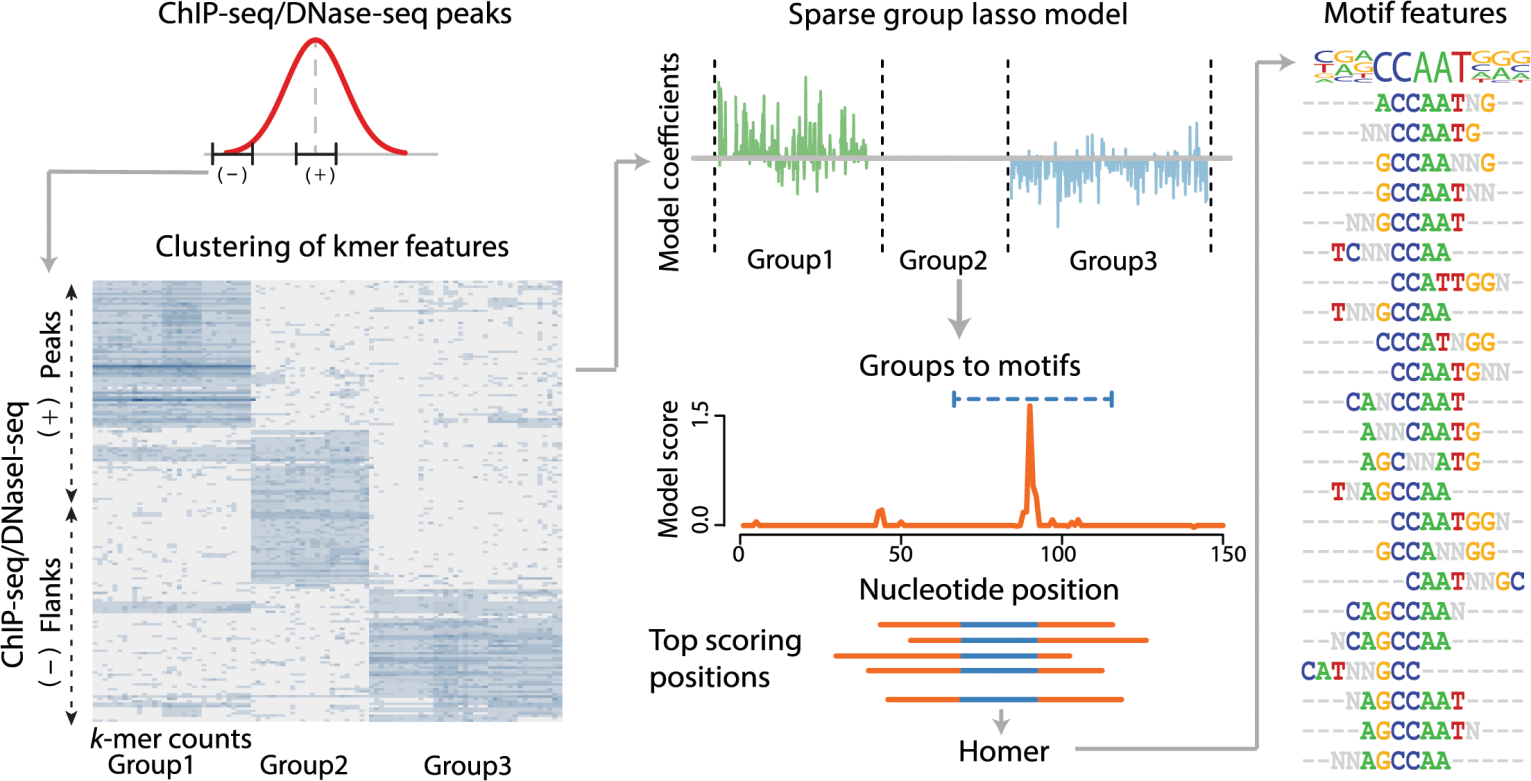
SeqGL identifies binding profiles in genome-wide regulatory maps. SeqGL uses sparse group lasso to identify the most important *k*-mer groups that discriminate between ChIP-seq/DNase-seq peaks and flanks. Hierarchical clustering of *k*-mer counts across peak and flank sequences reveals a block structure that defines *k*-mer groups. A representative heatmap of *k*-mer frequencies for a subset of peaks and flanks is shown. Sparse group lasso regression sets some groups uniformly to zero; groups with non-zero weights define group signals that may represent binding sequence signals for individual TFs. Significant hits for each group signal are identified, and sequence windows around these hits are extracted. HOMER is then applied to the windows to associate these group signals with motifs for visualization and identification.

Here we present a new and flexible discriminative learning tool called SeqGL (Fig 1) that uses group lasso regularization [13] to identify multiple context-dependent TF binding signals from a single ChIP-, DNase-, or ATAC-seq profile. SeqGL does *not* search for instances of known TF motifs but rather learns binding signals *de novo* from the profile. These binding signals are based on weighted *k*-mer scoring and can be summarized as motifs and compared to known TF motif databases; however, SeqGL has the potential to discover novel motifs or distinct variants of known motifs. In extensive benchmarking experiments on ENCODE TF ChIP-seq data, we show that SeqGL outperforms widely used motif discovery methods both for the discriminative task of distinguishing TF ChIP peaks from flanking sequences and for cofactor signal detection. Further, SeqGL successfully scales to the complexity of regulatory signals in DNase-seq or ATAC-seq profiles, identifying numerous TF binding signals in DNase- or ATAC-mapped regulatory regions that are confirmed by ChIP-seq. Finally, we show how SeqGL can be trained in a multi-task setting, where we jointly train on experiments from multiple cell types in order to identify shared and cell-type specific binding signals or encode information about genomic context, such as gene proximity or chromatin state, into the task structure to reveal more detailed regulatory sequence information.

## Results

SeqGL identifies TF sequence signals underlying ChIP-seq or DNase-seq/ATAC-seq peaks by training a discriminative model based on *k*-mer features on peaks (positive examples) versus their flanks (negative examples), building on our previous efforts in learning discriminative models of TF binding preferences [14, 15] (Fig 1). We use a *k*-mer-based feature representation related to the wildcard kernel [16] for the learning framework (Materials and Methods). Hierarchical clustering of these features across peak and flank sequences reveals a block structure, identifying subsets of *k*-mers that co-occur in subsets of examples. Thus we encode these *k*-mer clusters or groups using a sparse group lasso constraint [13] in a logistic regression model, which assigns non-zero weights to *k*-mer groups that significantly discriminate between peaks and flanks while setting other groups uniformly to zero (Materials and Methods). We view each non-zero *k*-mer group as the potential binding signal of a particular TF. In order to associate each group signal with the TF motif for visualization and identification, we first determine examples that are significantly discriminated by the *k*-mer group using an empirical null distribution and extract sequence windows containing the significant hits (Materials and Methods). We then use an existing motif algorithm (HOMER, [5, 17]) to generate a motif from these windows. Note that the motifs identified by HOMER are used for visualization and comparison to existing motif databases and not for prediction of binding sites. Thus SeqGL predicts multiple TF binding profiles for a DNase-seq/ATAC-seq or ChIP-seq experiment corresponding to *k*-mer group signals, along with associated motifs and significant hits. TFs are organized as structural families that often share a motif or have very similar motifs in existing databases. Therefore, SeqGL typically associates each non-zero *k*-mer group with the motif of a TF family rather than a specific factor. In analyses and validations presented below, we used existing ChIP-seq data or mRNA expression when available to resolve the specific factor of the family.

### SeqGL outperforms existing motif algorithms for discriminating ChIP-seq peaks

**Fig 2:**
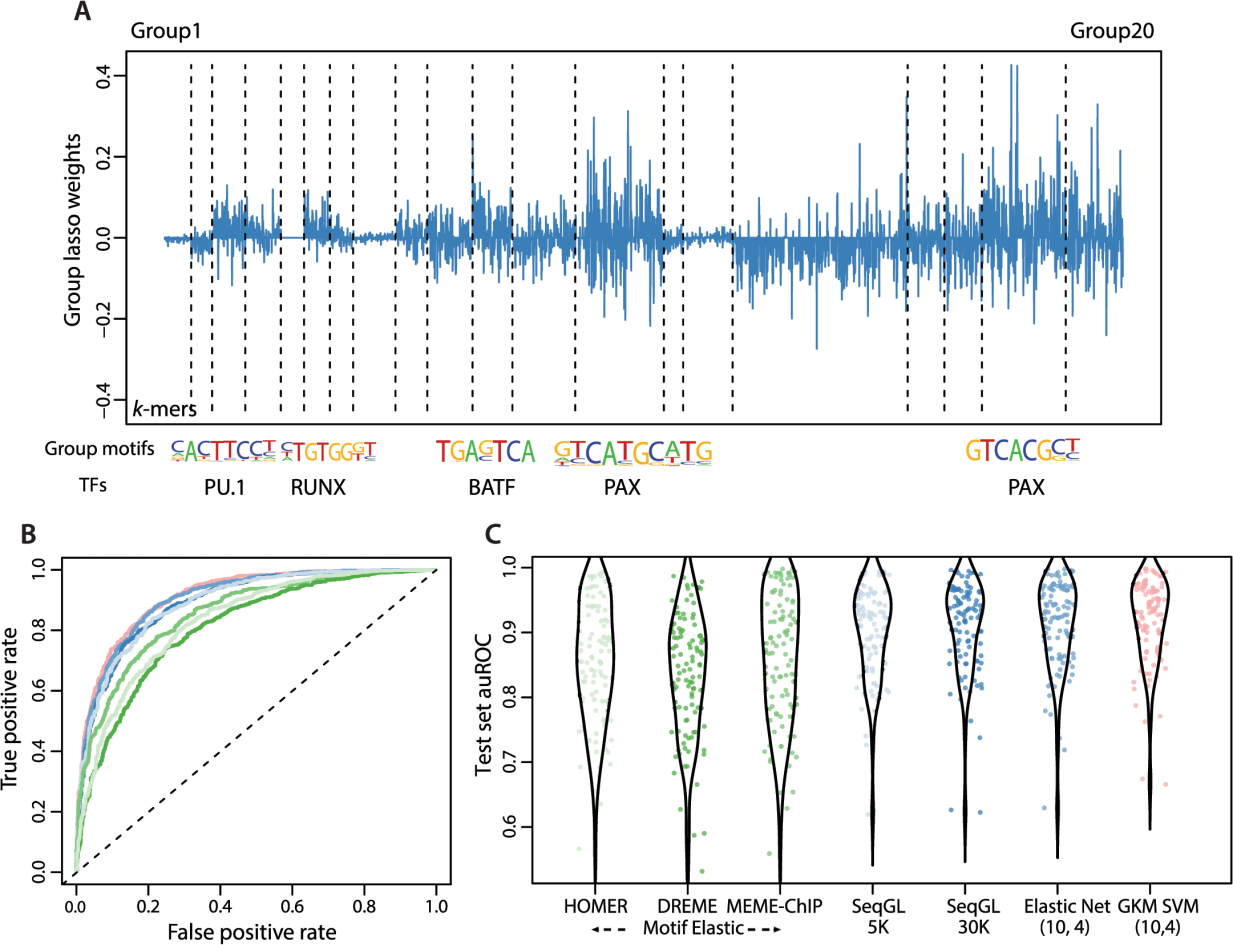
SeqGL performs significantly better than traditional motif discovery methods across different settings. (A) Plot showing the *k*-mer weight inferred group lasso regularized logistic regression for PAX5 ChIP-seq in GM12878 cell line. A number of groups are uniformly set to 0 (Group 5), while other groups are either significantly predictive of peaks or flanks (Group 3 and Group 2 respectively). Motifs identified for groups that are strongly predictive of peaks and the corresponding TFs are also shown. (B) PAX5 ChIP-seq auROC on the test set comparing the discriminative performance of SeqGL with motif finding tools and *k*-mer methods. The different colors correspond to the colors in Fig 2C. (C) Plots showing auROCs on test sets for 105 ChIP-seq experiments using different tools and settings. Three different settings were used for the motif finding tools HOMER, DREME and HOMER (see S1 Fig). ‘Best motif’ uses the highest-ranking motif from each method, as defined by the *p*-value; ‘Max motif’ uses the motif with maximum log odds score in each example; and ‘Motif elastic’ uses elastic net logistic regression across all motifs determined by the respective method. Only the ‘Motif elastic’ methods are shown in the performance plots, since they outperform the ‘Best motif” and ‘Max motif” methods. ‘SeqGL and other *k*-mer methods significantly outperform the different motif finding tools across all settings (Wilcoxon rank sum *p*-values < 7e-3). gkm-SVM performs marginally (but not significantly) better compared to SeqGL with 5K top discriminative features (Wilcoxon rank sum *p*-value = 0.06); SeqGL using a larger feature set (30K) gives identical performance to gkm-SVM (no difference in the distribution of auROC scores based on a Wilcoxon rank sum test, using *p*-value < 0.05 for our threshold of significance). Furthermore, the elastic-net regressor on the full SeqGL feature space using 10-mers with 3 wildcards (similar to settings used by gkm-SVM) also yields identical performance. While the discriminative accuracy is comparable, unlike other *k*-mer methods, SeqGL identifies multiple distinct DNA binding signals from the same ChIP-seq experiment (S3 Table).

We compared the performance of our method for the task of discriminating peaks from flanks to a number of widely used motif finding tools: HOMER (a PSSM-based approach designed for TF ChIP-seq data) [5], DREME (a *k*-mer-based discriminative motif tool in the MEME suite) [4], and MEME-ChIP (an EM-based motif tool) [6]. Our benchmark dataset consisted of 105 different ENCODE [18] ChIP-seq experiments across two cell lines: GM127878, a lymphoblastoid cell line, and H1-hESC, an embryonic stem cell line. We used the multiple motifs identified by each tool in different settings to compare the performance. “Best motif” uses PSSM scores from the best motif identified by the tool for each example (mean auROC for HOMER:. 775, DREME:. 747 and MEME-ChIP:. 777). “Max motif” uses the maximum log odds score of any motif for each example (mean auROC for HOMER:. 739, DREME:. 781 and MEME-ChIP:. 738). We found that SeqGL performs significantly better than all the tools in both these settings (Wilcoxon rank sum *p*-values < 7e-3) (S1 Fig; mean auROC for SeqGL:. 921). We note that all methods find the “known” motif in almost the same number of experiments (S2 Table); therefore, the performance advantage of SeqGL derives in part from combining multiple signals. For this reason, we also trained a “Motif elastic” model for each motif discovery tool, using elastic net logistic regression [19] with the PSSM scores for all motifs as features. (Materials and Methods). Motif elastic is the method most comparable to SeqGL since we expect each *k*-mer group in SeqGL to represent binding preferences of a particular transcription factor. We note that the “Motif elastic” is the best performance setting for each motif tool, and yet SeqGL significantly outperforms “Motif elastic” for all tools (Fig 2, mean auROC for HOMER:. 858, DREME:. 846 and MEME-ChIP:. 876). This result demonstrates the advantage of representing binding signals as weighted *k*-mer scoring models and learning these signals at the same time as the peaks-vs-flanks classifier.

### SeqGL retains the discriminative advantage of SVM k-mer kernel methods

Several other *k*-mer based discriminative models have recently been proposed to learn TF binding preferences from ChIP-seq data, including two SVM methods: the di-mismatch kernel, based on *k*-mer features in the dinucleotide alphabet counted with mismatches [14]; and the gkm-SVM method [20], which is very similar to the wildcard kernel introduced some time ago [16]. Importantly, both these kernel methods represent the binding model as a “bag of *k*-mers”, which does not allow obvious extraction of multiple distinct binding signals. Nevertheless, both methods were able to outperform single motif methods for the statistical task of discriminating peaks from non-peaks in held-out examples from the training ChIP-seq experiment, and the gkm-SVM (similar to the wildcard kernel) computes the kernel over *all k*-mers with a fixed length and number of wildcards and trains in the dual space. Therefore, it is worth comparing to these approaches to confirm that our group lasso regularization in the primal space of a reduced set of *k*-mers still retains the advantage of previous kernel methods.

For our method comparison, we used SeqGL both with the default 5K features (8-mers with up to two consecutive wildcards) and with 30K features. We compared to the di-mismatch kernel using 5K features and published parameters and to gkm-SVM using 10-mers with up to 4 wildcards; we also performed a simple elastic net regularization with logistic regression on the set of 10-mer features with up to 4 wildcards (Fig 2). When we evaluated performance differences between SeqGL (5K features) and other *k*-mer methods with a Wilcoxon rank sum test, no method significantly outperformed SeqGL, while SegGL did have a significant win over the di-mismatch kernel (median auROC of. 921 versus. 884, *p* < 2e-10, Wilcoxon rank sum test); when we used all di-mismatch features, its performance improved (median auROC of. 906) to a statistical tie with SeqGL. The gkm-SVM method obtained a slightly higher median auROC of. 931, but the performance difference compared to SeqGL was not statistically significant (*p* = 0.06, Wilcoxon rank sum test). When we increased SeqGL to retain 30K *k*-mer features, performance improved (mean auROC of. 927), giving a statistically tie with gkm-SVM and elastic net on 10-mer features with up to 4 wildcards. All the *k*-mer based methods outperformed all the motif elastic methods (*p* < 3e-4, Wilcoxon rank sum test, for all pairwise comparisons). We therefore concluded that SeqGL, even with shorter *k*-mers and only 5K features, achieved statistically equivalent discriminative performance to more computationally expensive kernel methods that use a much larger implicit feature space.

### SeqGL identifies multiple TF binding signals in ChIP-seq profiles

**Fig 3:**
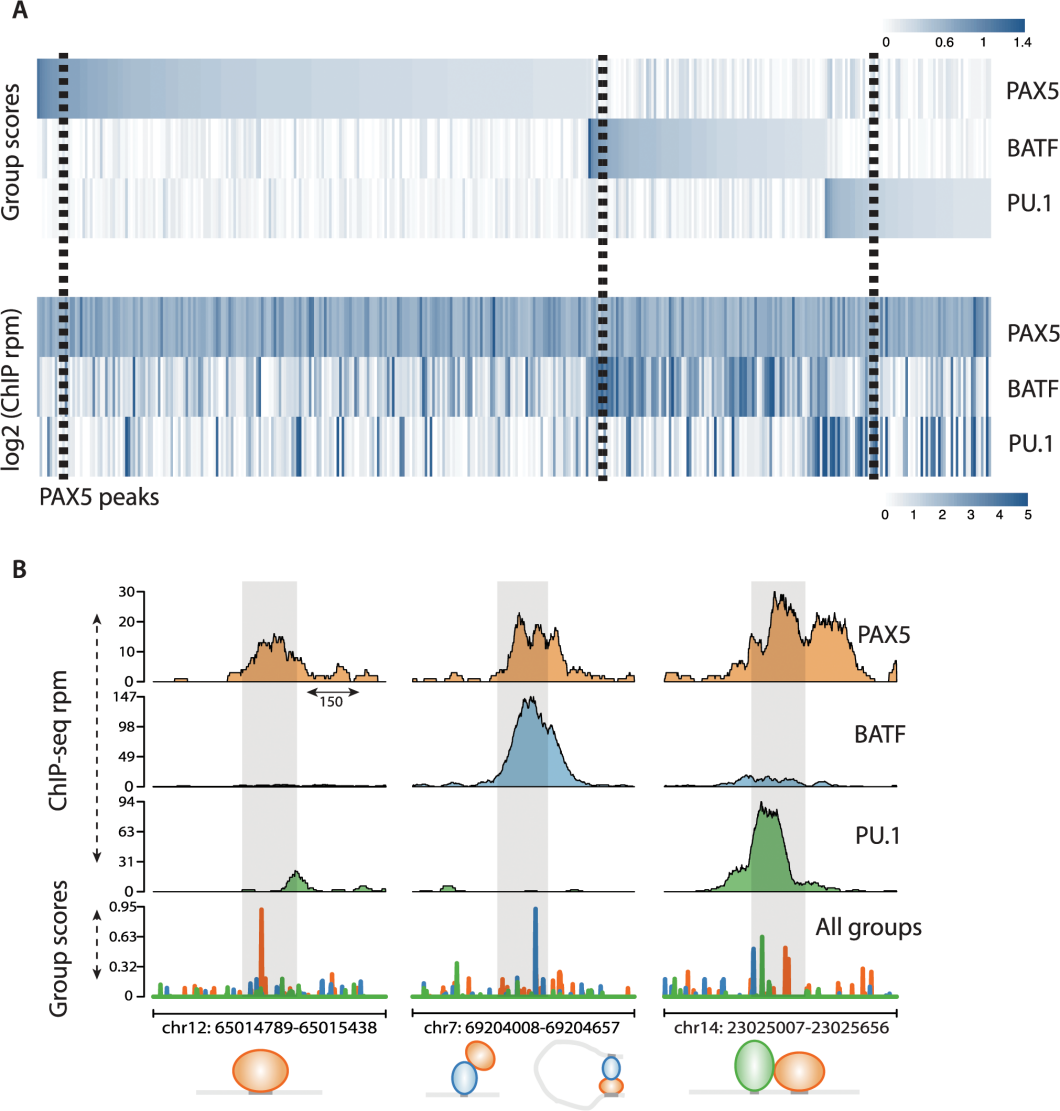
SeqGL identifies binding signals in ChIP-seq occupancy profiles. (A) Heatmaps show predicted binding signals and ChIP occupancy of PAX5 and co-factors. SeqGL analysis of PAX5 ChIP-seq predicts BATF and PU.1 as the most significant binding partners of PAX5. The top panel shows the group scores associated to three TFs from the PAX5 model, and the bottom panel shows the corresponding ChIP-seq read counts. This shows that a number of PAX5 ChIP-seq peaks are indirect and obtained through DNA binding of partners rather than PAX5 itself. The dashed boxes highlight the specific examples illustrated in Fig 3B. (B) Specific examples of PAX5 profiles show various modes of binding detected by SeqGL. The left panel shows direct binding of a TF (PAX5 recognizing its motif). The middle panel shows that the sequence signal is associated to BATF, and hence the PAX5 peak at this location is either due to interaction of the two factors and/or long distance looping. The right panel shows an example of co-binding of PAX5 and PU.1, each recognizing its respective binding motif.

PAX5 is an important B cell lineage factor expressed at early stages of B cell differentiation [21]. Therefore we used PAX5 ChIP-seq data in GM12878 to examine the co-factor binding profiles identified by SeqGL (Fig 3). SeqGL was run with 20 groups and identified 7 groups to be significantly predictive of peaks compared to flanks. The top panel of Fig 3A shows the group scores for three highest scoring groups ranked by their predictive power of peaks compared to flanks, and the bottom panel shows the ChIP-seq read counts for the corresponding TFs. While SeqGL identified 7 groups as predictive of PAX5 peaks, we are highlighting the three highest ranked groups for simplicity. As expected, the top-scoring group in the PAX5 ChIP-seq experiment identifies sites that are strongly associated with the canonical PAX5 motif. The other groups are associated with AP family and PU.1 motifs, which have prominent roles in B cell function [21]. We next used existing ChIP-seq data to validate these predictions (S2 Fig, Materials and Methods) and found that PAX5 peaks predicted by these two groups are indeed bound by AP family factors and PU.1 respectively. Furthermore we also identified BATF as the specific AP factors since peaks associated with this group are most enriched for BATF ChIP-seq peaks. Interestingly, even though the PAX5 ChIP-seq read densities are uniform across all peaks, the group scores show significant differences, and a significant number of PAX5 peaks do not have a sequence signal for PAX5. We propose that this observation is due to different modes of binding. Fig 3B shows specific examples of these modes of binding. The left panel shows the direct binding mode: a TF recognizing its canonical motif. The middle panel shows that even though there is a strong PAX5 peak, the sequence signal is actually derived from a different factor, BATF, indicating either indirect PAX5 binding via a protein-protein interaction or potentially a distal looping interaction. This illustrates that for a region with ChIP-seq peaks for multiple factors; the binding signals need only come from a subset of those factors. Finally the right panel demonstrates co-binding of PAX5 and PU.1 with each factor recognizing its respective motif. These observations are consistent with the different modes of interaction between TFs identified by Wang et al. [8]. The fraction of peaks with non-canonical signal is dependent on the TF; we observed a continuous spectrum across ChIP-seq experiments, with some TFs showing exclusively canonical signals and others showing a mix of canonical and non-canonical signals (S3 Fig and S3 Table). We note that gkm-SVM has a procedure for producing PSSMs from the top ranked *k*-mers in the model and reports three motifs per TF ChIP-seq experiments. However, when we compared results with SeqGL and HOMER, we saw that gkm-SVM missed many of the co-factor signals identified by SeqGL and indeed often returned three variants of the same motif (S4 Table).

### SeqGL reveals genomic context dependent and cell-type specific binding signals

**Fig 4:**
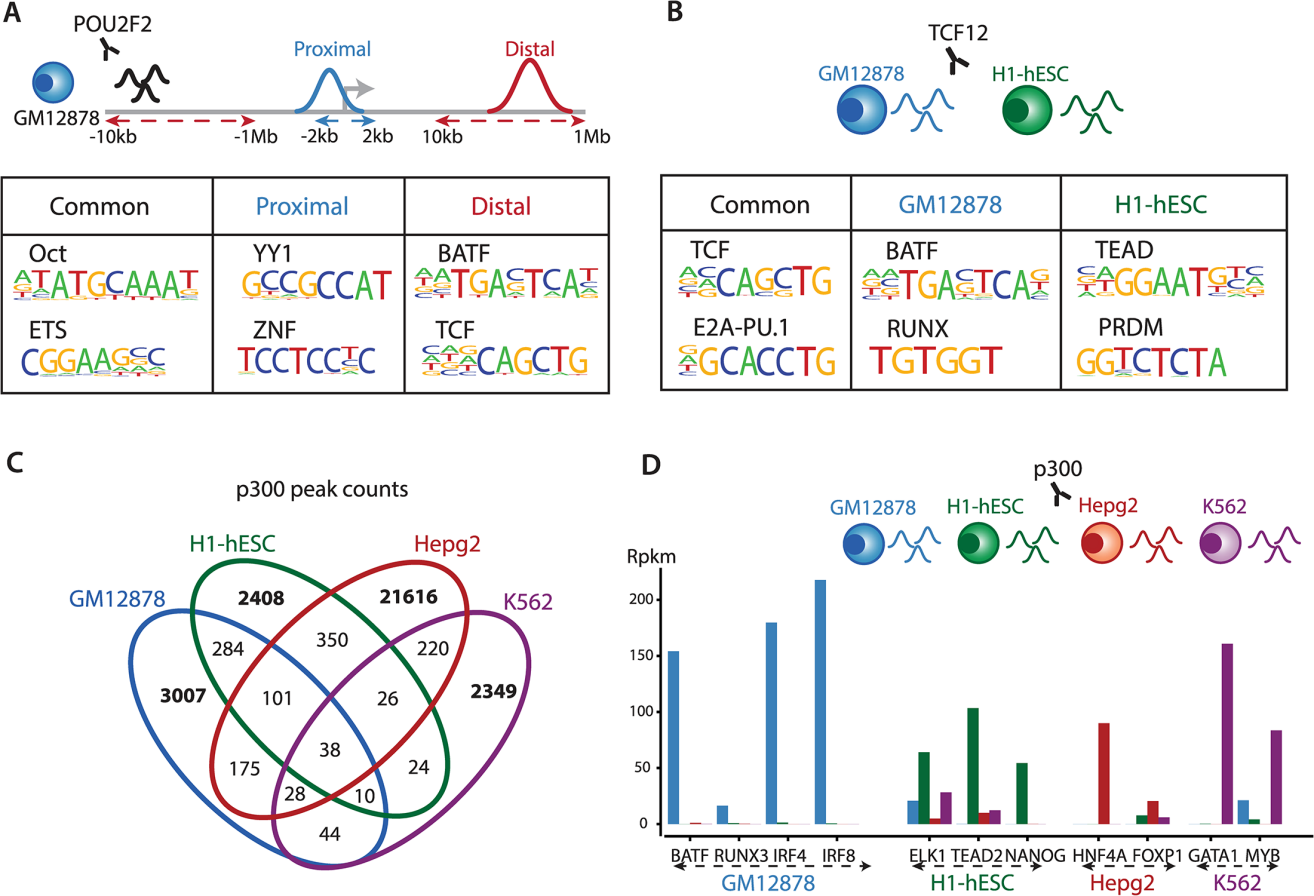
Candidate binding partners for a transcription factor are dependent on genomic context, cell type, and expression levels of partners. (A) Multitask learning identifies different candidate binding partners of POU2F2 at proximal and distal locations to genes. The proximal partners are associated with factors that preferentially bind to CG-rich motifs whereas the distal partners are associated with cell-type specific regulators. As expected, the POU/octamer motif is present in both contexts. (B) Similarly, TCF12 binding profiles in GM12878 and H1-hESC shows preference for cell-type specific regulators at the cell-type specific sites, and the TCF motif is associated with peaks of both cell types. (C-D) The enhancer bound factor p300 shows highly cell-type specific binding and predicted TF signals. The cell-type specific expression of the p300 candidate binding partners partially explains the differing binding profiles of p300 across cell types.

We next used SeqGL to examine the connection between binding context and sequence signals in TF occupancy profiles. To this end, we used a multitask technique [22] to identify gene proximal and distal binding profiles of POU2F2, a B cell maturation factor [23]. Briefly, proximal and distal peaks are considered two different classification tasks for multitask learning. This formulation combines the peaks of the two tasks to create a third task also called the “common” task. All the three tasks are solved simultaneously to identify factors that are not only common to both tasks but also specific to each task i.e., context independent as well as context-specific factors (Materials and Methods, S4 Fig). Fig 4A illustrates that as expected the octamer motif representing the OCT TF family is strongly associated with both proximal and distal peaks, as is the motif for ETS, which binds at both proximal and distal sites. Interestingly, the proximal sites show a strong association with CG-rich motifs whereas the distal sites are associated with factors like BATF and TCF, which are necessary for B cell function [21]. This is consistent with the observation that cell type information is encoded at distal enhancers rather than proximal promoters [24]. Note that the motifs for YY1, ETS, ZNF and TCF families were detected specifically by SeqGL and not by HOMER. On a similar note, we used the same multitask technique to encode the cell type context of TCF12 by joint training on ChIP-seq data for this factor in both GM12878 and H1-hESC (Fig 4B). As expected, the TCF motif is associated with peaks common to both cell types whereas the candidate binding partners are completely different and are key regulators of the particular cell type (BATF and RUNX for GM12878; TEAD and PRDM for H1-hESC).

The expression levels of binding partners may play an important role in determining the context for binding of certain transcription factors. In both the POU2F2 and TCF12 analysis, the enhancer and cell-type specific profiles respectively had cell-type specific transcription factors as candidate binding partners. To explore this further, we applied SeqGL to the binding profiles of the enhancer binding factor p300 in different cell types. p300 peaks across different cell types are largely cell-type specific (Fig 4C) and the binding profiles are enriched for factors that are expressed specifically and thus functionally relevant in the respective cell type (Fig 4D). Please note that the specific factors were identified using mRNA expression for groups associated with TF families. BATF, IRF4 and IRF8 show GM12878-specific expression; TEAD4 and NANOG, genes that play a central role in embryonic stem cells [25], are specifically expressed in H1-hESC along with ELK1; HNF4A, a gene necessary for liver development [26], is specifically expressed in HepG2, a hepatocellular carcinoma cell line; and finally GATA1, which is involved in myeloid development [27], and MYB are specifically expressed in K562, a myelogenous leukemia cell line. These results demonstrate that beyond the DNA sequence signal, the cell type and genomic context of binding for a particular TF may define its binding partners, and furthermore the expression of potential binding partners can lead to altered binding profiles.

### SeqGL improves sensitivity for detecting binding signals in DNase-seq profiles

SeqGL is particularly effective for determining TF binding profiles in DNase-seq data since DNase peaks contain signals for a large multiplicity of transcription factors. Our group lasso approach is well suited to capture this diversity of sequence signals in DNase peaks. We used DNase-seq data from GM12878 because of the availability of an immense collection of ChIP-seq experiments in this cell type. As a first step, we used MACS [28] to identify broad DNase peaks, followed by PeakSplitter [28] to identify subpeaks within the broader peaks (S5 Fig). We then used IDR [29] to identify a robust set of reproducible subpeaks across replicates (Materials and Methods).

**Fig 5:**
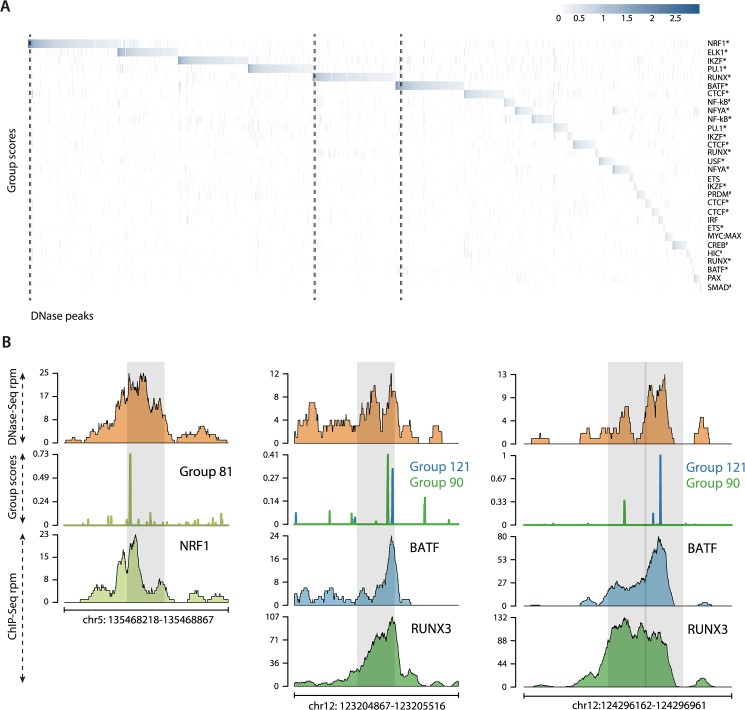
Sequence preferences of GM12878 DNase-seq peaks. (A) Heatmap showing the group scores for top groups over 16,891 GM12878 DNase-seq peaks. Note that some peaks have a sequence signal for a single factor while others have signals for multiple factors (FDR-corrected *p* < 0.01). All the group predictions identified by “*” have been validated by ChIP-seq data, while “#” indicates no ChIP-seq data available in ENCODE. The dashed boxes highlight the specific examples illustrated in Fig 5B. (B) The left panel shows a DNase peak with a strong score for a single transcription factor (NRF1). The middle panel shows DNase peaks with moderate scores for both BATF and RUNX. The left panel shows different binding preferences in adjacent split peaks derived from a single broad peak. All the predictions are validated by ChIP-seq.

Fig 5A shows the predicted TF binding profiles of broad DNase peaks in GM12878 after summarizing the group scores over subpeaks. A total of 16,891 peaks are shown with group scores for top 30 groups. 68/200 groups are associated with DNase peaks and contain motifs for 38 TFs (S5 Table). A number of groups are associated with motif variants of the same TF. HOMER and MEME-ChIP were unable to identify 14/38 motifs (S5 Table). While HOMER is able to find motifs that are significantly enriched in the peaks, SeqGL specifically identified motifs for TFs such as EBF1, E2A, and SOX4, which are important for B cell function but present in a smaller fraction of DNase peaks. Furthermore we validated 37/46 groups with ENCODE ChIP-seq data (indicated by “*” in Fig 5A). There is a strong enrichment for transcription factors with known function in B cell identity and activity (S6 Table). Closer inspection of the group scores revealed that a subset of peaks (9262 out of 34303) have a signal for a single transcription factor (Fig 5B, left panel) whereas a larger subset (20048 out of 34303) have sequence signals for multiple factors (FDR-corrected *p* < 0.01) (Fig 5B, middle panel). Note that the BATF-RUNX pattern is one of the many strongly appearing co-binding patterns (S7 Table). This observation is similar to the results from PAX5 ChIP-seq (Fig 3B), where a PAX5 peak is not necessarily accompanied by an underlying PAX5 motif. Furthermore subpeaks identified from a single broad peak can have sequence signals for different groups/factors (Fig 5B, right panel) highlighting the value of splitting broad peaks into their constituent components. Thus SeqGL learns extensive regulatory sequence information from DNase-seq by predicting binding profiles for multiple TFs and identifying their combinations. Furthermore, a number of groups are associated with motifs that only partially match to known motifs indicating that these are either variants of existing motifs or potentially novel motifs that have not been characterized (S6 Fig).

### SeqGL generalizes to ATAC- mapped regulatory regions

**Fig 6:**
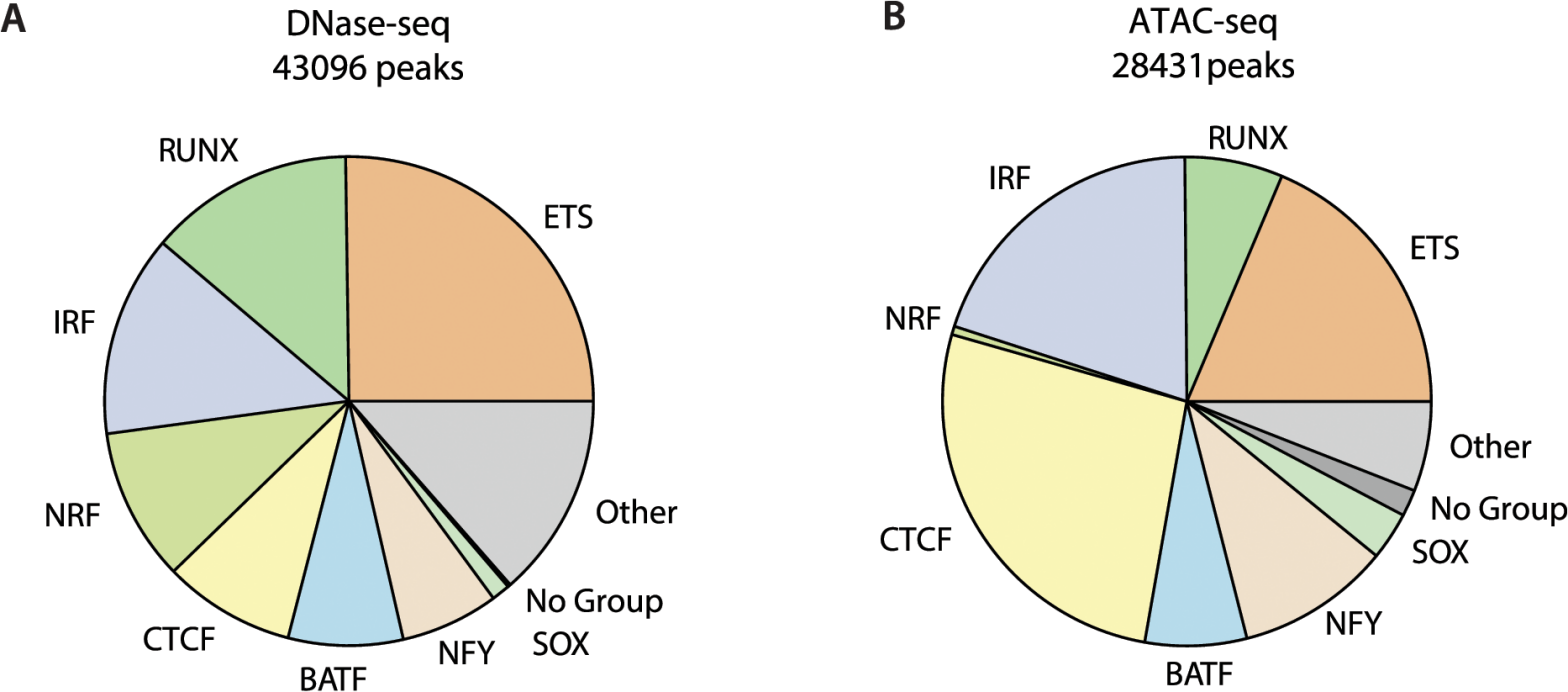
SeqGL identifies sequence signals underlying ATAC-seq peaks. Distribution of top-scoring TFs across (A) DNase-seq and (B) ATAC-seq peaks. TFs identified by SeqGL using ATAC-seq peaks are IRF, BATF and other cell-type factors that are strongly represented across peaks in both data types whereas NRF and CTCF show enrichment in DNase-seq and ATAC-seq respectively. The fraction of intergenic enhancer peaks is significantly higher in ATAC-seq, potentially explaining the higher occurrence of CTCF.

We further assessed the ability of SeqGL to identify binding profiles for multiple TFs using the recently developed ATAC-seq assay in GM12878, an alternative approach for mapping regions of open chromatin that can be performed on 500–50,000 cells [30]. Using the same settings as for DNase-seq peaks, SeqGL identified 30 group signals associated with ATAC-seq peaks. Corresponding TF ChIP-seq data is available for 23 group signals, and we were able to validate the predictions for 18 of these 23 groups (S8 Table). The TFs identified in ATAC-seq peaks are primarily a subset of TFs identified using GM12878 peaks with the more frequent TFs identified in both datasets. Fig 6 shows the distribution of maximum scoring TFs for DNase-seq (Fig 6A) and ATAC-seq peaks (Fig 6B). Cell type factors like BATF, IRF and RUNX are strongly represented in both datasets whereas the promoter binding TF NRF and insulator protein CTCF have strong enrichment in DNase-seq and ATAC-seq peaks, respectively. Interestingly, the fraction of intergenic peaks is significantly higher in ATAC-seq compared to DNase-seq (42% in ATAC-seq compared to 33% in DNase-seq). This difference in TF signal distribution is also present in peaks common to DNase-seq and ATAC-seq (S7 Fig), suggesting that the assay-dependent training set alters the relative strengths of the group signals for different TFs.

### SeqGL identifies context-specific sequence signals in DNase-seq profiles

**Fig 7:**
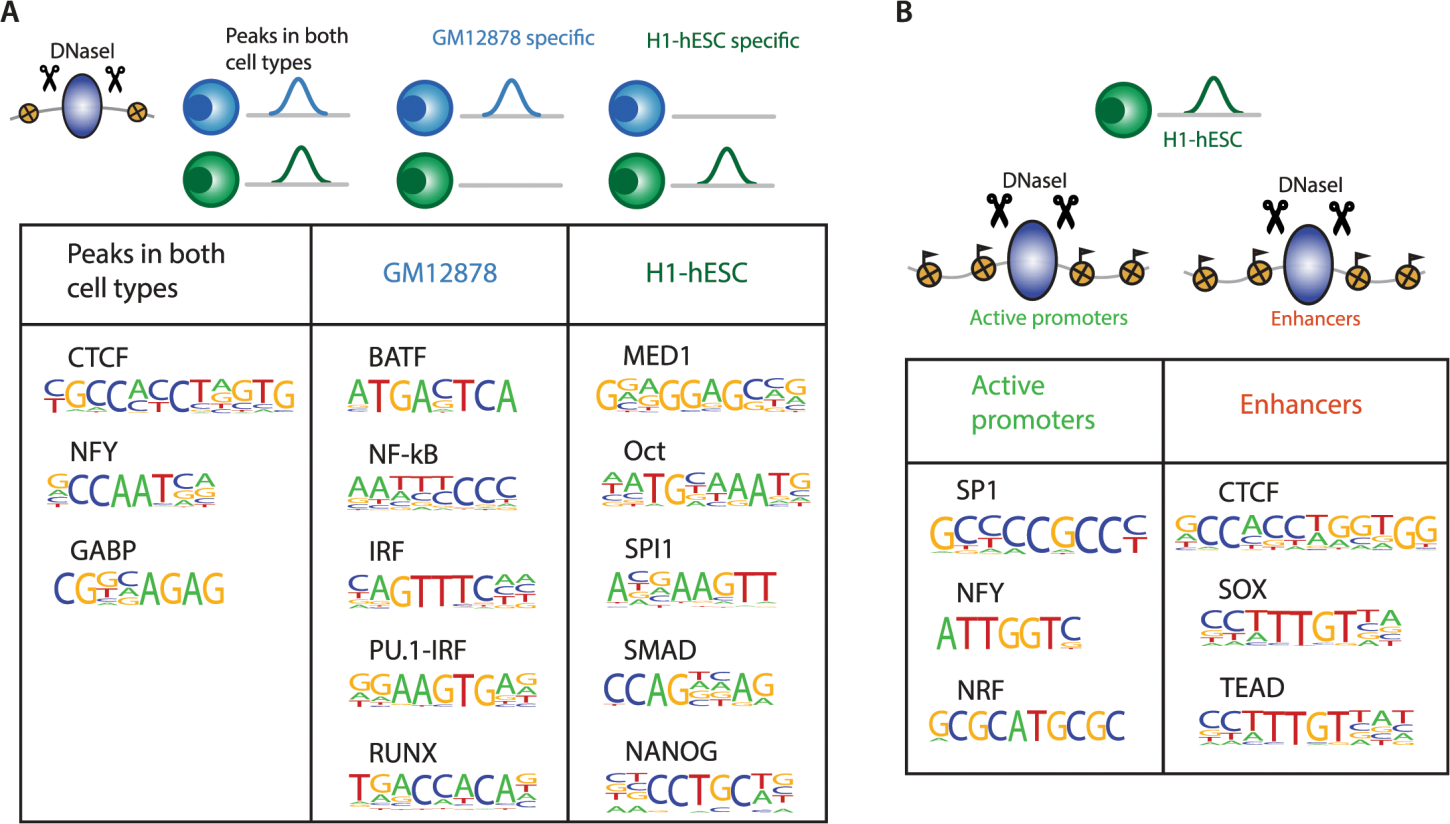
Context determinants of DNase binding signals. (A) DNase peaks across cell types show significant differences in the underlying sequence preferences depending on the context. DNase peaks that are common to both GM12878 and H1-hESC cell lines show strong preferences for either insulator proteins (CTCF) or promoter associated regulators (NFY) whereas again cell-type specific peaks show preferences for cell-type specific regulators (BATF, IRF, RUNX in GM12878 and OCT, SMAD, NANOG in H1-hESC). (B) The chromatin context of a DNase peak also defines a context for specific binding preferences. DNase peaks in active promoter regions are associated with NRF, SP1 and NFY motifs whereas peaks in the enhancer regions are associated with CTCF, SOX and TEAD motifs.

Cell type and chromatin state both provide the context determinants for the TF binding profiles underlying mapped regulatory elements. For example, while a number of DNase peaks are cell-type specific, a significant fraction show comparable accessibility in multiple cell types (S8 Fig). We built binding profiles for DNase peaks common to both GM12878 and H1-hESC and peaks specific to the two cell types (Materials and Methods). As expected, the cell-type specific profiles are associated with cell-type specification transcription factors: BATF, IRF, PU.1 and RUNX in GM12878 and OCT4, SPI1 and NANOG in H1-hESC (Fig 7A). Intriguingly, peaks that are common to both cell types contain not only promoter-associated factors like NFY but also the insulator protein CTCF. This appearance of insulator/structural proteins was consistently observed in many comparisons and thus may indicate that the broader domains of regulation remain consistent across different cell types [31]. Furthermore, we also explored the chromatin context in the H1-hESC cell line. Using ENCODE ChromHMM segments [32], we predicted TF binding profiles for DNase peaks in active promoters and enhancers. Active promoters as expected contained CG-rich and motifs for TFs such as NFY and SP1 that are known to bind promoter regions whereas, interestingly, enhancers are associated not only with cell-type specification factors but also with CTCF (Fig 7B). This result suggests that both cell-type specification factors and structural proteins are needed to build the enhancer landscape of cells.

## Discussion

The use of *k*-mers for the representation and discovery of regulatory motifs has a long history. Several early papers used over-represented *k*-mers to identify TF binding sites and other sequence signals (e.g. [33, 34]), and methods like Weeder and MITRA organized efficient searches for enriched *k*-mers and composite *k*-mer patterns, respectively, using traversal of suffix tree or retrieval tree data structures to count occurrences of *k*-mer occurrences with inexact matches [35, 36]. The first *k*-mer based string kernels were introduced shortly afterwards [37, 38] for the problem of SVM classification of protein domains and used the same retrieval tree data structure for efficient kernel computation of *k*-mer features with mismatches, wildcards, or gaps [16]. However, it was also recognized in those early years that *k*-mer based SVMs could be used to model regulatory sequences in DNA and RNA. The original application in this domain was for extracting intronic splicing silencers and enhancers [39], and subsequently *k*-mer kernel methods were introduced for recognition of alternatively spliced exons, gene structure prediction, and nucleosome positioning [40–42], where in each case, the discriminative *k*-mer model captured subtle regulatory sequence signals.

With the advent of large-scale *in vitro* and *in vivo* TF binding assays, discriminative learning of TF binding preferences using discriminative *k*-mer methods became feasible. The first study of this kind introduced the di-mismatch kernel with SVR and SVM models to learn TF binding preferences from protein binding microarray (PBM) and TF ChIP-seq data [15], and this model was later used to systematically examine the cell-type specificity of TF sequence preferences using large-scale ChIP-seq data from two ENCODE cell lines [14]. Other studies adapted previous *k*-mer kernels to train discriminative models on TF ChIP-seq data [20, 43] or used *k*-mer features with lasso regularization to investigate sequence signals associated with histone marks [44]; many *k*-mer methods were also benchmarked in a DREAM competition for learning TF binding models from PBM data, though the focus of this study was clearly to compare methods generating PSSMs [45]. Indeed, despite the 10-year history of *k*-mer based discriminative learning methods and several previous reports that these methods outperform traditional motif discovery for the *statistical* problem of discriminating TF ChIP-seq peak from non-peak sequences [14, 20], it is inarguable that traditional PSSM methods are far more widely used than *k*-mer based methods in the larger genomics and biology communities. It is therefore worth asking what is the essential limitation of *k*-mer methods, as they exist in the literature, that prevents their more widespread adoption.

Here we propose that the key limitation of existing *k*-mer based discriminative methods is the difficulty of determining what information the model is using to achieve its improved performance, extracting these sequence signals from the model, and using them to dissect the regulatory code. We are indeed aware of only one previous method that tries to interpret the sequence information from a *k*-mer based discriminative model, the POIM (positional oligomer importance matrix) framework for weighted degree kernels [46], and this method requires that examples are aligned to each other and assumes at most a single “motif” per position. In our setting, a discriminatively trained *k*-mer model for TF ChIP-seq data may be capturing subtle preferences of the ChIP-ed TF, co-factor signals, and general compositional biases associated with regions of open chromatin—all of which are biologically interesting sequence signals but are difficult to deconvolve from a standard *k*-mer kernel SVM. These sequence signals also depend on cellular context, suggesting that we should be cautious about touting performance on the purely statistical problem of predicting occupancy on held-out peaks/non-peaks from the training experiment. From a practical standpoint, this is an artificial problem, as genome-wide occupancy in the training experiment is already known; the more meaningful question is how well the discriminative model can explain occupancy in a distinct cell type, so that a new ChIP-seq experiment need not be done. This problem necessitates an investigation into what the TF binding model is capturing, and how well we might expect it to generalize to a new cellular context. In our previous di-mismatch work [14], we showed that for some TFs, discriminative *k*-mer models indeed capture cell-type specific TF binding preferences, which we were able to interpret as variants in the binding model of the ChIP-ed TF. What were unable to do what do cleanly extract co-factor signals that may also account for cell-type specificity of TF occupancy. A more recent study using the gkm-SVM method [20] (similar to the wildcard kernel [16]) presented a heuristic for extracting PSSMs from the SVM but also largely missed co-factor signals. This gap in the literature motivated the current work.

SeqGL represents a new methodology for deciphering *multiple* binding sequence signals in epigenomic data sets. It combines discriminative learning on a wildcard *k*-mer representation with group lasso regularization to retain the better accuracy of *k*-mer based methods compared to traditional motif discovery algorithms while achieving greater sensitivity for identifying multiple sequence signals. Furthermore, the framework scales to handle the large multiplicity of TF binding signals in DNase-seq data. Through multitask training; SeqGL can identify TF binding signals that are common or specific to different genomic contexts or cell types. The use of structured constraints in the primal space (here based on group lasso) together with multitask learning provides the necessary framework to disentangle multiple constituent signals associated with context-specific regulatory information. As such, we believe that SeqGL represents an important advance for using discriminative *k*-mer methods to address biologically meaningful questions in regulatory genomics. SeqGL is available as an open source R package at http://cbio.mskcc.org/public/Leslie/SeqGL/.

## Materials and Methods

### Data and preprocessing

ChIP-seq and DNase-seq data was downloaded from ENCODE [18]. We used a total of 105 TF ChIP-seq experiments for the GM12878 and H1-hESC cell lines (S1 Table). Peaks called by ENCODE were used for ChIP-seq analysis and histone context was identified using the ENCODE-defined ChromHMM segments. ENCODE data accession numbers: GSE32465, GSE31477, GSE29692.

### DNase-seq peak calling

We first pooled DNase-seq data for all replicates of a given cell type and identified peaks using MACS [28] with a low threshold of FDR-corrected *p* < 1e-3 using the Benjamini-Hochberg procedure for multiple hypotheses correction. The broader peaks identified by MACS were then split into smaller peaks, to localize binding of a single TF, using PeakSplitter [28]. Reproducible peaks were then identified using IDR [29] at a threshold of 0.01 in every pairwise replicate comparison (S9 Fig). We identified a total of 43,105 subpeaks spanning 34,303 peaks in GM12878 and 102,349 subpeaks spanning 78,180 peaks in H1-hESC.

### ATAC-seq data processing

ATAC-seq data was processed using the procedure described for DNase-seq data. We used fragments of length < = 100 for peak calling and downstream analysis.

### Gene expression

We downloaded the RNA-seq bam files for all cell types from ENCODE. We used the *summarizeOverlaps* function from the GenomicRanges Bioconductor package [47] to count the reads mapping per gene. We used these counts to determine mean RPKM values for each gene across replicates for a cell type.

### SeqGL

#### Model overview

SeqGL performs a classification task between peaks and flanks to identify sequence preferences of transcription factors and their binding partners (Fig 1). We use *k*-mer-features rather than known motifs to capture these sequence preferences. We use a window of 150 bp around the peak summit as positives and a 150 bp window, 300 bases upstream of the peak summits as negatives in the classification task. We assume that the sequence signal for the transcription factor is present in this window. Our feature representation is related to the wildcard kernel [16], where we use all *k*-mers of length 8 with up to two consecutive wildcards to define features. Note that we use all exact-matching *k*-mers and *k*-mers with wildcards as separate features. A *k*-mer and its reverse complement are treated as a single feature and include the counts of the *k*-mer and its reverse complement.

We then use hierarchical clustering of these *k*-mer features using Spearman correlation as a distance metric to identify clusters/groups of non-overlapping *k*-mers underlying subsets of examples. We chose 20 groups with 5000 top discriminative features between peaks and flanks for ChIP-seq experiments and 200 groups with 30K top discriminative features for DNase-seq experiments by empirical testing. These groups are then encoded in a group lasso constraint in the classification problem. We use logistic regression as our classification tool with sparse group lasso constraints [13] to identify the most discriminative groups of *k*-mers.

This is defined by the objective function:

$${\text{Min}}_{w}\sum_{i}\text{log}\left(1+\text{exp}\left(-y_{i}w\cdot x_{i}\right)\right)+\lambda_{1}\sum_{g}\sqrt{l_{g}}{\left\|w_{g}\right\|}_{2}+\lambda_{2}\sum_{m}|w^{m}|$$

where **x**_*i*_ represents the *k*-mer count vector for example *i*; *y*_i_ are the labels: +1 for peaks and -1 for flanks; **w**_g_ = (*w* ^*1*^, *w* ^*2*^ *… w* ^*lg*^) is the vector of *k*-mer weights of group *g*; *l*_*g*_ is the number of *k*-mers in group *g* and *w* ^*m*^ is the weight of *k*-mer *m*. The first summation is over all the examples (peaks and flanks) and defines the logistic loss function. The second summation encodes the group lasso constraint across all *k*-mer weights **w**_*g*_ for all groups *g*. The third constraint encodes the sparsity constraint over all *k*-mers. The two regularization parameters *λ*_*1*_ and *λ*_*2*_ control sparsity at the group level and *k*-mer level respectively [13]. We used the SPAMS toolbox for solving the classification problem [48], and *λ*s were chosen by 10-fold cross-validation on the training sets.

#### Class scores and significant hits

We determined a ranking of groups using the following scoring scheme. For each group, we identify a “positive class score” and “negative class score” which measures the contribution of the group to identify positive and negative examples respectively.

$$score(g,i)=\text{log}\left(1+\text{exp}\left(-y_{i}w_{g}\cdot x_{i,g}\right)\right)$$

$$score\left(g,class\right)=\sum_{i\in class}score\left(g,i\right)$$

We associate the group with either the positive or negative class depending on which score is the maximum. Next we identify peaks that are significantly defined by each group. For a positive class associated group *g*, we use the group scores of all the negative examples of *g* as the empirical null distribution. We used a Benjamini-Hochberg procedure to identify group members at a 5% false discovery rate (S10 Fig). We use groups with at least 25 examples for motif finding. Similarly for a group associated with the negative class, the scores of the positive examples are used as the empirical null. This group membership scheme can be applied to any set of sequences to predict binding of specific TFs.

#### Groups to motifs

We use the HOMER motif-finding tool [5] to associate each group with a motif. We first identify the maximum scoring position within the 150 base span for each group member. We then use a 50 base window around this position for finding motifs using HOMER (Fig 1). Thus we use subregions of sequences for a subset of examples to find the motif underlying each group. The statistics of the group size are primarily a function of the diversity of sequence signals in a given experiment. As an example, we find that most of the training and test peaks for EBF1 have the canonical motif, and therefore most of informative *k*-mers are clustered into a single group.

The SeqGL tool is available as an R package at http://cbio.mskcc.org/public/Leslie/SeqGL/. A complete table of candidate binding partners for different ChIP-seq experiments can be found at http://cbio.mskcc.org/public/Leslie/SeqGL/chip_results/index.html.

### Performance comparison

We compared the performance of SeqGL to a number of motif finding tools: HOMER, DREME and MEME-ChIP. We analyzed 105 different ChIP-seq experiments from the GM12878 and H1-hESC cell lines (S1 Table) and used area under the Receiver Operating Curve (auROC) on the test set as the performance measure. We determined the top 2000 peaks in each ChIP-seq experiment and split them evenly into training and test sets. auROCs were determined for the test sets after using the training set for learning the model or motifs. The same training and test sets were used for SeqGL, HOMER and DREME. A first order Markov model was estimated from the negative sequences in the same training set for MEME-ChIP. Note that increasing the number of peaks used for training and test does not significantly alter performance (S11 Fig). We also tested SeqGL using dinucleotide shuffled sequences as negatives instead of sequences in the flanks (S12 Fig). This leads to significantly better performance (*p* < 2e-7, Wilcoxon rank sum test), demonstrating that shuffled sequences are relatively “easy” negatives and therefore not a strong adversary. Dinucleotide shuffled sequences also lead to better performance compared to HOMER and MEME-ChIP in the “Motif elastic” setting (S11 Fig; *p* < 2e-10, Wilcoxon rank sum test). Elastic net was performed using the glmnet R package [19].

### Context dependent partners: ChIP-seq

We used regularized multitask learning as proposed by Evgeniou & Pontil [22] (S3 Fig) to learn context-specific candidate binding partners for POU2F2 and TCF12 ChIP-seq (Figs 4A and 4B). In addition to the two tasks (POU2F2: (a) Proximal peaks vs flanks & (b) Distal peaks vs flanks and TCF12: (a) GM12878 peaks & (b) H1-hESC peaks respectively), we defined a “common” task that solves both the tasks as a single classification problem. The common task is expected to capture the information common to both the tasks (POU2F2 and TCF12 binding preferences respectively) whereas the task specific model will capture the context-dependent binding partners. We ran clustering for each task separately to identify task specific groups. We then learned models for all these tasks using the multitask learning formulation below. Let

$$L\left(x_{i},y_{i},t_{i}\right)=\text{log}\left(1+\text{exp}\left(-y_{i}\left(w_{c}+w_{t_{i}}\right)\cdot x_{i}\right)\right)$$

represent the logistic loss, and the objective function can be defined as

$${\text{Min}}_{w_{c},w_{T_{1}},w_{T_{2}}}\sum_{i}L\left(x_{i},y_{i},t_{i}\right)+\lambda_{1}\left(\alpha \sum_{t\epsilon \left\{t_{1},t_{2}\right\}}\sum_{g}{\left\|w_{t,g}\right\|}_{2}+\beta \sum_{g}{\left\|w_{c,g}\right\|}_{2}\right)+\lambda_{2}\left(\alpha \sum_{t\epsilon \left\{t_{1},t_{2}\right\}}\sum_{m}\left|w_{t}^{m}\right|+\beta \sum_{m}\left|w_{c}^{m}\right|\right)$$

where **w**_*c*_, **w**_*T1*_ and **w**_*T2*_ are the common, first task, and second task models respectively. *L(* **x**_*i*_, *y*_*i*_, *t*_*i*_ *)* is the logistic loss defined for each example belonging to task *t*_*i*_. The second component of the equation encodes the group lasso constraints for all group models, and third component encodes the sparsity constraint with **w**_*t*,*g*_ representing a vector of *k*-mer weights which belong to group *g* in task *t* and *w*_*t*_^*m*^ representing the *m*-th *k*-mer weight in task *t*. *α* and *β* trade off between the task specific and common task components. We used *α =* 1.5 and *β* = 1 which gave us the best test performance.

### Validation of binding predictions by ChIP-seq

We validated the binding partner prediction using existing ChIP-seq data from ENCODE. We again used the auROC measure for validation (S1 Fig). We first identified the overlaps of the training set with the peaks of a particular TF. These overlapping peaks are considered positive examples and the non-overlapping training peaks as the negative set. We then used the group scores for each sample as predictions to determine the auROC. We consider a motif prediction validated if the predicted TF or a member of the TF family is in top five auROC predictions. Many of the cases that are not validated show only marginally matching motifs to the TF under consideration. In these cases, the derived motif and known TF motif are dissimilar to each other, and therefore the associated TF might not necessarily be correct. We resolve the ambiguity among TF family members by using the expression levels of different family members in the cell type or the ChIP-seq experiment with the best auROC in this validation. As an example, BATF is identified as co-factor of PAX5 since Group 11 is best predicted by BATF and not other members of the AP.1 family, and RUNX3 is identified in GM12878 DNase-seq since RUNX3 is expressed at significantly higher levels compared to other RUNX family members.

### Context dependent partners: DNase-seq

We learned all the models separately for identifying context-dependent binding signals using DNase-seq data. We used DNase peaks falling completely within specific ChromHMM states for learning the results in Fig 7B. The ChromHMM state “Active Promoters” was used for promoter peaks and “Weak Enhancers” and “Strong Enhancers” states for the enhancer peaks.

## Supporting Information

S1 Fig Comparison of SeqGL to different motif and *k*-mer methods. We compared the discriminatory power of SeqGL to three widely used motif finding tools: (1) HOMER, (2) DREME and (3) MEME-ChIP. Multiple motifs are identified by each method and we use three different settings for comparison: “Best motif” uses the PSSM score from the best motif identified by the tool, “Max motif” uses the maximum log odds score of any motif for each example and “Motif elastic” uses elastic net logistic regression with PSSM scores for all motifs as features. SeqGL with 5K top discriminative features outperforms these methods across all the three settings (Wilcoxon rank sum *p*-values < 7e-3). We also compared the results of SeqGL to different *k*-mer kernels: SeqGL with standard 5K features significantly outperforms the publish di-mismatch kernel which used 1K features (Wilcoxon rank sum *p*-value < 2e-10) and di-mismatch with all features performs more comparably (Wilcoxon rank sum *p*-value < 0.3). SeqGL with 30K features gives comparable performance to the recently described gkm-SVM (Wilcoxon rank sum *p*-value < 0.4). The performance is again similar using the SeqGL features with different *k*-mer lengths and wildcards using elastic net regression.
(PDF)

S2 Fig Validation of co-factor sequence signal prediction by ChIP-seq. Example plot showing receiver-operating curves for validation of co-factor signal prediction. SeqGL associates the BATF motif with Group 8 in the IRF4 ChIP-seq experiment. For each TF with ChIP-seq data, the corresponding peaks overlapping with the IRF4 peaks are considered as positive examples and those not overlapping as negative examples, and the group scores are used as a ranking to determine the ROCs. The top three TFs as ranked by auROCs are shown in the plot. The red line represents the ROC for BATF, blue and green for JUND and FOXM1, respectively. The best auROC for IRF4 Group 8 is BATF thus validating the BATF motif prediction.
(PDF)

S3 Fig Canonical and non-canonical sequence signals in ChIP-seq experiments. A number of TFs show strong presence of the canonical sequence signal in a majority of peaks. At the other end of the spectrum some TFs are not associated with the canonical sequence signal in any of the peaks. These include TFs like STAT (S4 Table).
(PDF)

S4 Fig Multitask representation. We use a multi-task learning framework to identify motifs common to both the tasks. The model of each task is then encoded as sum of task specific and common models. The features of the two tasks are stacked to create a common feature matrix and define groups for each of set of features separately.
(PDF)

S5 Fig Identification of subpeaks. Peak callers like MACS [28] identify broad peaks in region of high DNase density. MACS identified the region shown in the plot as one single peak. We used the PeakSplitter tool to identify constituent subpeaks and treated them as separate examples in our learning model. The split peaks are often associated with different TF motifs (Fig 2B).
(PDF)

S6 Fig SeqGL identifies novel motifs and variants of existing motifs. A number of groups in GM12878 DNase-seq analysis are associated with motifs that only partially match to known motifs. These motifs are potentially novel motifs that have not been characterized or variants of known motifs.
(PDF)

S7 Fig TF distribution among peaks common to DNase-seq and ATAC-seq peaks. The enrichment of NRF and CTCF motifs in DNase-seq and ATAC-seq respectively is also observed in peaks common to both datasets. We identified common peaks by finding peaks that overlap by at least 90% after defining windows of 150 bases around the peak summits.
(PDF)

S8 Fig Shared and differential DNase peaks for GM12878 and H1-hESC. The scatterplot of log read counts of DNase peaks between two cell types shows both a large number of cell-type specific peaks as well as common peaks. DESeq [49] was used to identify the cell-type specific and common peaks. FDR corrected *p*-value of 0.01 was used for cell-type specific peaks whereas peaks with FDR corrected *p*-value > 0.25 were used as common peaks. The peaks in green and blue are used as H1-hESC and GM12878 specific peaks and peaks in black are used as common peaks for identification of motifs in Fig 2C.
(PDF)

S9 Fig Reproducible DNase peaks using IDR. We use IDR with a cutoff of 0.01 to identify reproducible subpeaks in each cell type. The plot shows the identified subpeaks in H1-hESC.
(PDF)

S10 Fig Identification of peaks associated with each group. Group members for the positive class are determined using the scores of the negative class as the empirical null distribution (black line). We calculate an FDR-corrected *p*-value for each positive peak using this empirical null. A 5% FDR threshold was used in the association of peaks with groups.
(PDF)

S11 Fig Increasing sample size does not affect SeqGL performance. In order to test the effect of increasing noise in the training set, we used (a) 5000 examples and (b) 10000 examples for training and test in ChIP-seq experiments where sufficient peaks are available. Increasing the number of training examples does not significantly affect performance either with 5000 examples (*p* < 0.6, Wilcoxon rank sum test) or 10000 examples (*p* < 0.25, Wilcoxon rank sum test)
(PDF)

S12 Fig Using dinucleotide shuffled sequences as negatives leads to significantly higher performance. (A) We used dinucleotide shuffled sequences instead of flanking sequences as negatives and observe a significantly better SeqGL performance with shuffled sequences (*p* < 2e-7, Wilcoxon rank sum test). Thus shuffled sequences are “easy” negatives and do not present a strong adversary. Moreover, SeqGL using dinucleotide shuffled sequences as negatives significantly outperforms HOMER and MEME-ChIP on this task (*p* < 2e-10, Wilcoxon rank sum test). (B) Plot showing distribution of nucleotide frequencies in the negative flank sequences across all 105 ChIP-seq experiments. This shows that negative flank sequences are not enriched for polyA sequences. We also enumerated the number of low complexity sequences in the dataset (a sequence was defined to be low complexity if a particular nucleotide is repeated in 50% of sequence positions). <2% of the flank sequences were identified as low complexity (as opposed to <1% of peak sequences) indicating that flank sequences are not enriched for low complexity sequences.
(PDF)

S1 Table List of ChIP-seq experiments. (XLSX)

S2 Table Identification of known TF motifs by SeqGL and HOMER. (XLSX)

S3 Table Canonical and non-canonical sequence signals. (XLSX)

S4 Table Cofactors identified by SeqGL, HOMER and gkm-SVM. (XLSX)

S5 Table TFs associated with each group and ChIP-seq validation for GM127878 DNase-seq peaks. (XLSX)

S6 Table Biological significance of TFs underlying GM12878 DNase peaks identified by literature search. (XLSX)

S7 Table GM12878 co-binding patterns. (XLSX)

S8 Table GM12878 ATAC-seq binding profiles. (XLSX)
