# Supplementary figures and images for: SeqGL Identifies Context-Dependent Binding Signals in Genome-Wide Regulatory Element Maps
Source: PLoS Comput Biol. 2015 May 27;11(5):e1004271. doi: 10.1371/journal.pcbi.1004271 (PMC4446265; doi:10.1371/journal.pcbi.1004271)

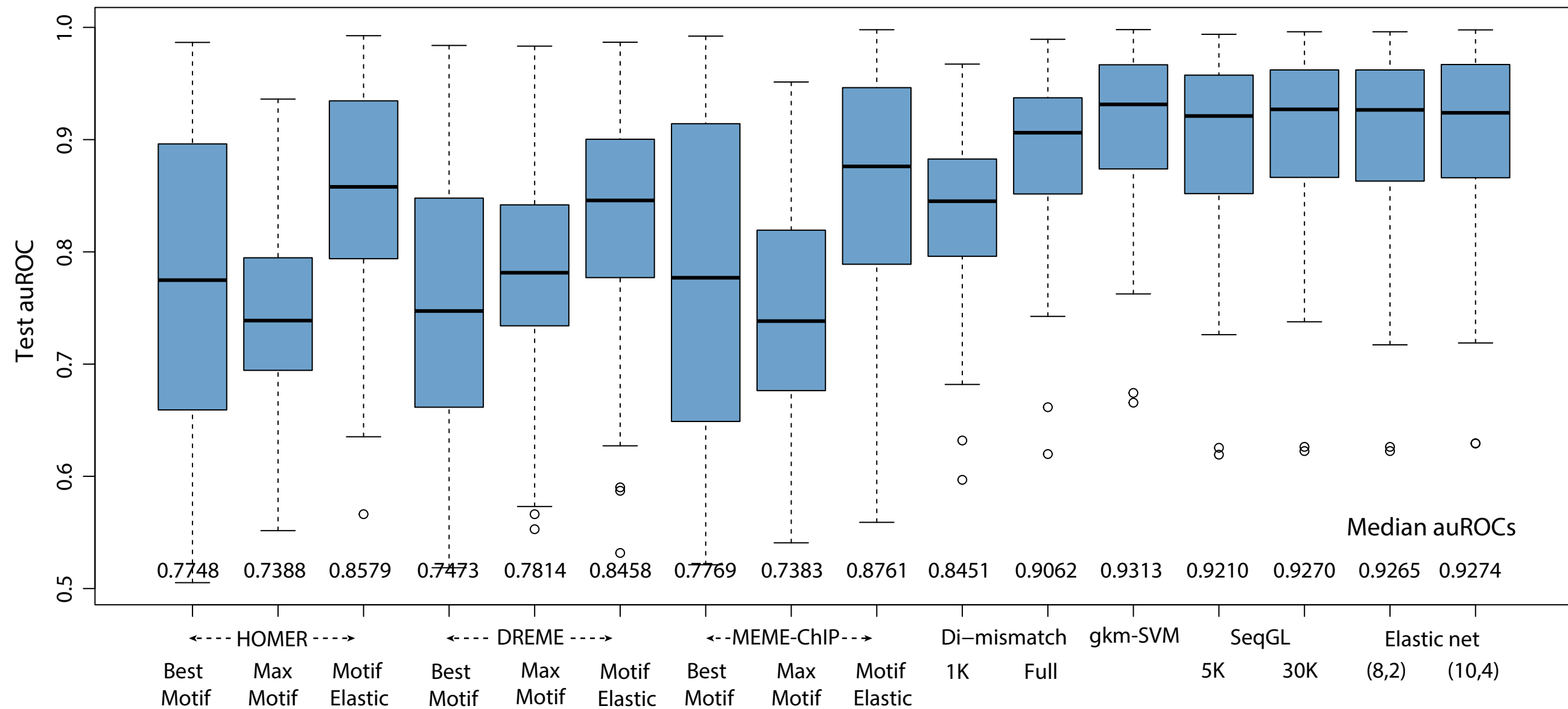

Supplement: S1 Fig — We compared the discriminatory power of SeqGL to three widely used motif finding tools: (1) HOMER, (2) DREME and (3) MEME-ChIP. Multiple motifs are identified by each method and we use three different settings for comparison: “Best motif” uses the PSSM score from the best motif identified by the tool, “Max motif” uses the maximum log odds score of any motif for each example and “Motif elastic” uses elastic net logistic regression with PSSM scores for all motifs as features. SeqGL with 5K top discriminative features outperforms these methods across all the three settings (Wilcoxon rank sum p-values < 7e-3). We also compared the results of SeqGL to different k-mer kernels: SeqGL with standard 5K features significantly outperforms the publish di-mismatch kernel which used 1K features (Wilcoxon rank sum p-value < 2e-10) and di-mismatch with all features performs more comparably (Wilcoxon rank sum p-value < 0.3). SeqGL with 30K features gives comparable performance to the recently described gkm-SVM (Wilcoxon rank sum p-value < 0.4). The performance is again similar using the SeqGL features with different k-mer lengths and wildcards using elastic net regression. (PDF) [file pcbi.1004271.s001.pdf]

## IRF4 Group 8

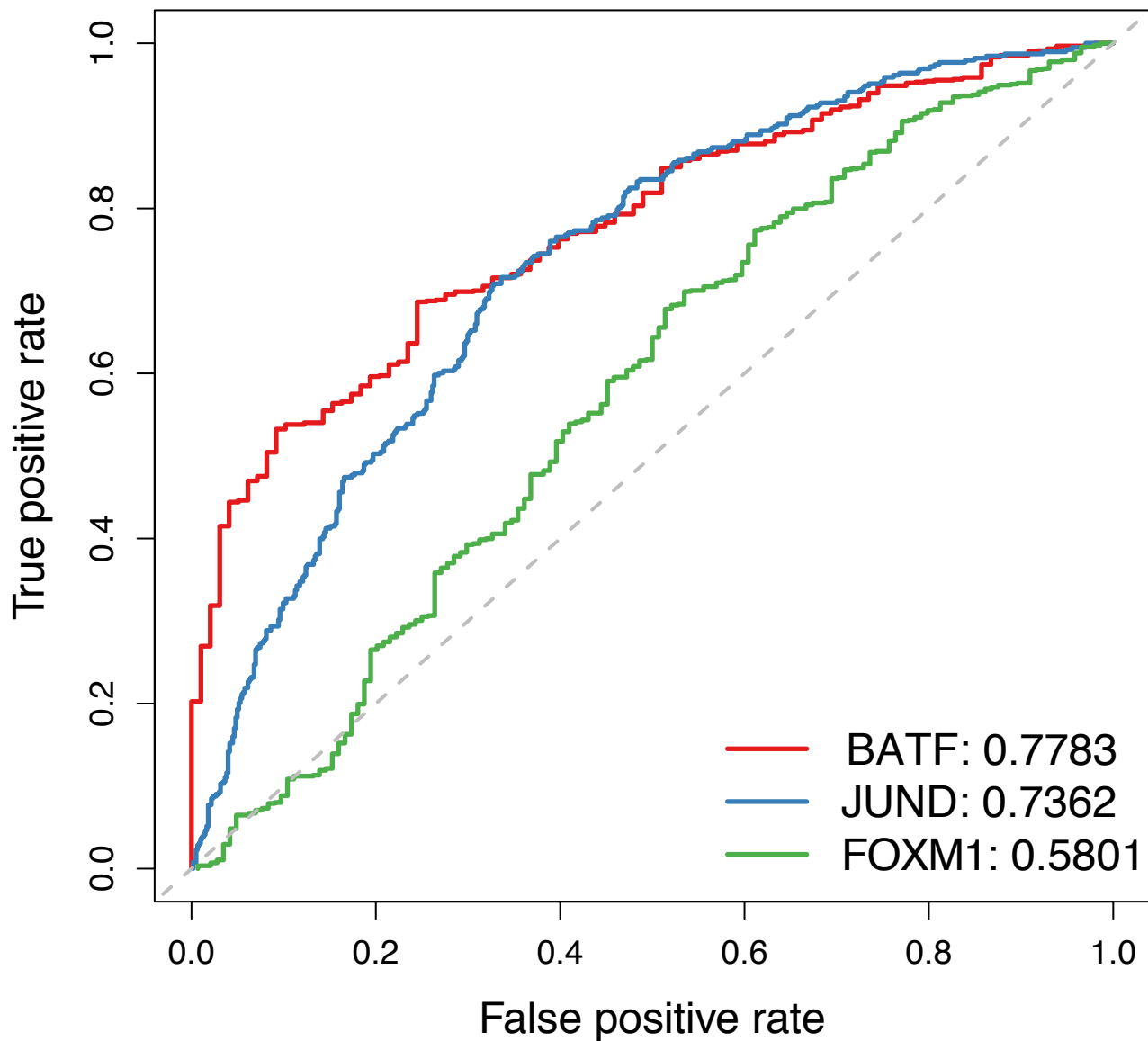

Supplement: S2 Fig — Example plot showing receiver-operating curves for validation of co-factor signal prediction. SeqGL associates the BATF motif with Group 8 in the IRF4 ChIP-seq experiment. For each TF with ChIP-seq data, the corresponding peaks overlapping with the IRF4 peaks are considered as positive examples and those not overlapping as negative examples, and the group scores are used as a ranking to determine the ROCs. The top three TFs as ranked by auROCs are shown in the plot. The red line represents the ROC for BATF, blue and green for JUND and FOXM1, respectively. The best auROC for IRF4 Group 8 is BATF thus validating the BATF motif prediction. (PDF) [file pcbi.1004271.s002.pdf]

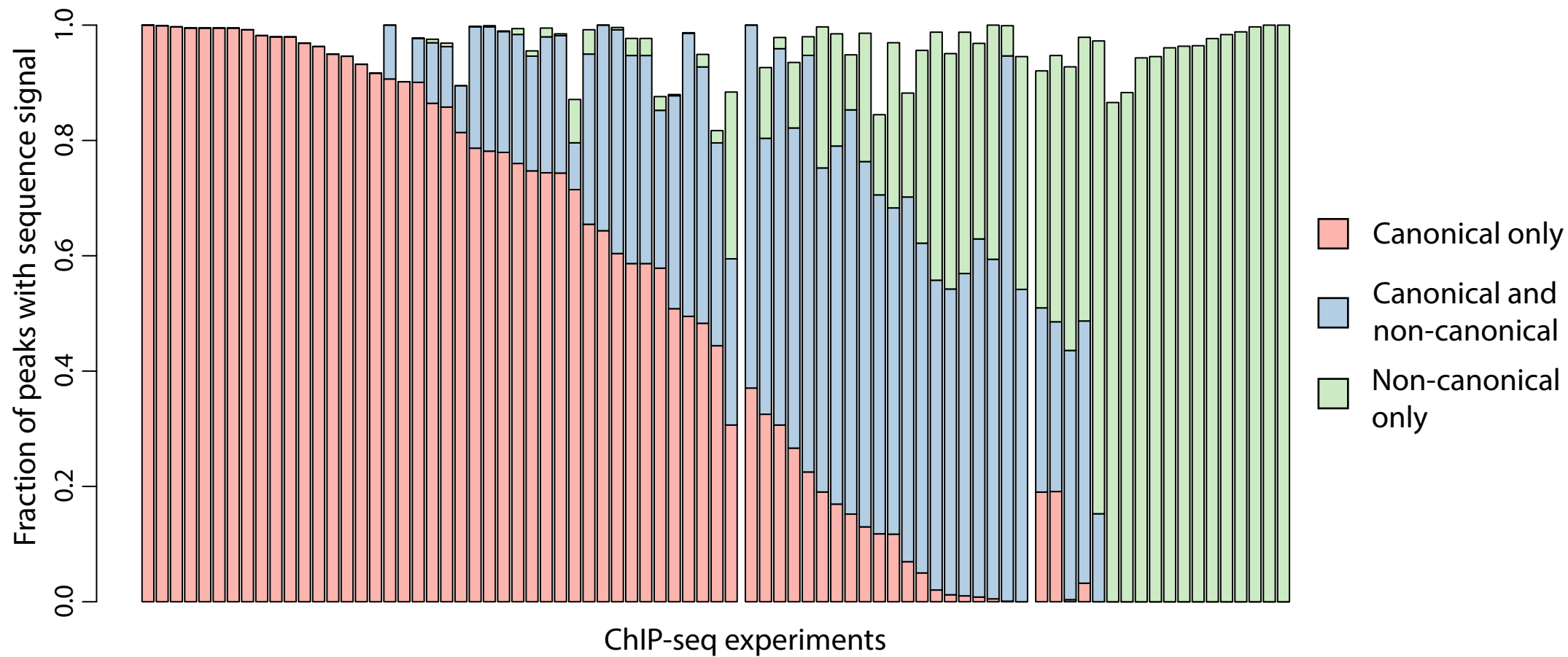

Supplement: S3 Fig — A number of TFs show strong presence of the canonical sequence signal in a majority of peaks. At the other end of the spectrum some TFs are not associated with the canonical sequence signal in any of the peaks. These include TFs like STAT (S4 Table). (PDF) [file pcbi.1004271.s003.pdf]

Common

Task1

Task2

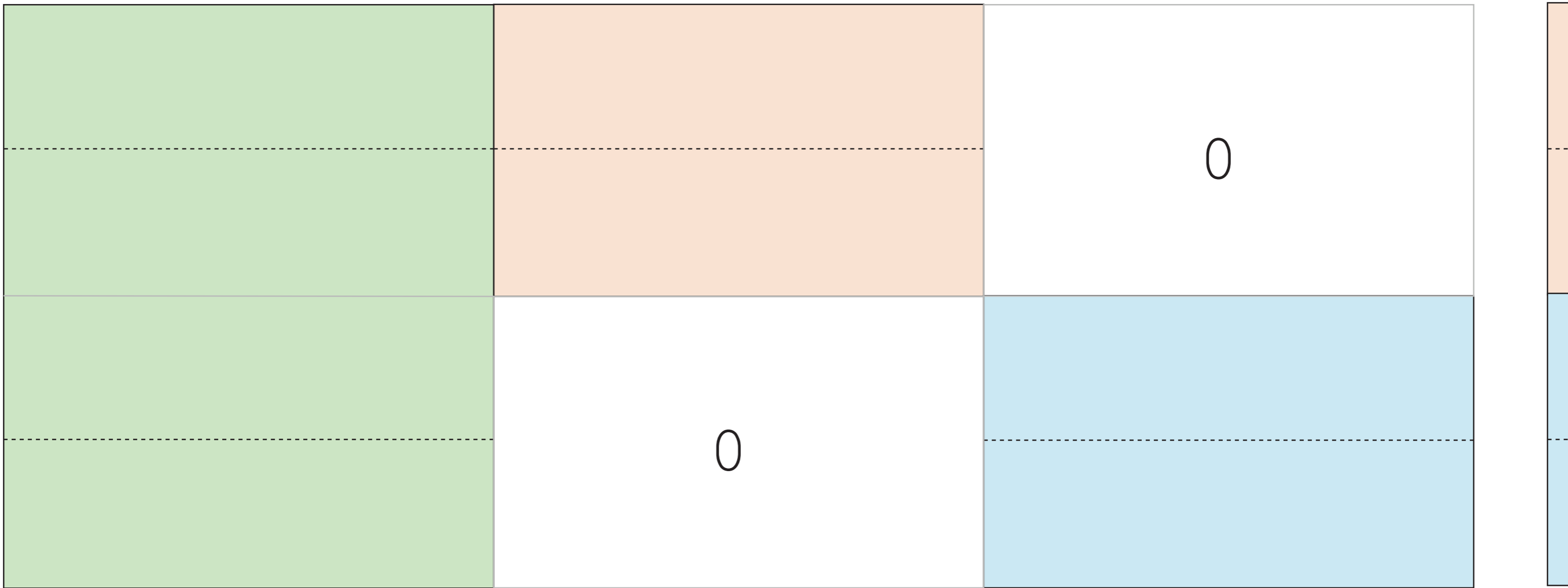

Supplement: S4 Fig — We use a multi-task learning framework to identify motifs common to both the tasks. The model of each task is then encoded as sum of task specific and common models. The features of the two tasks are stacked to create a common feature matrix and define groups for each of set of features separately. (PDF) [file pcbi.1004271.s004.pdf]

DNase-seq reads

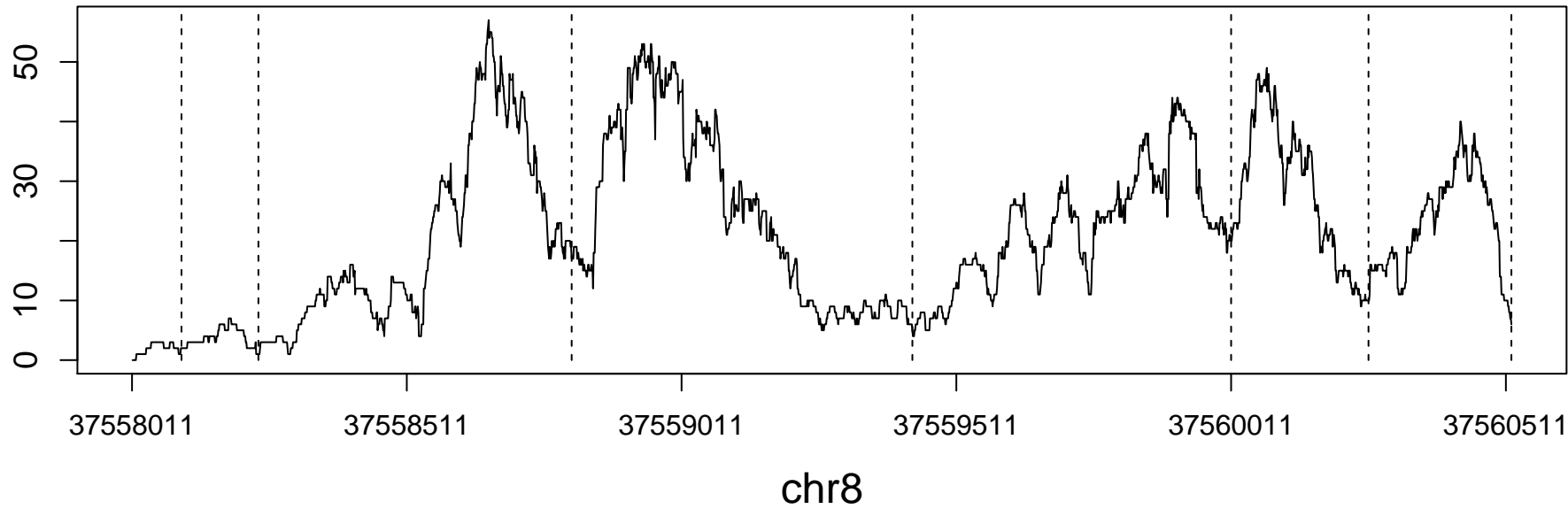

Supplement: S5 Fig — Peak callers like MACS [28] identify broad peaks in region of high DNase density. MACS identified the region shown in the plot as one single peak. We used the PeakSplitter tool to identify constituent subpeaks and treated them as separate examples in our learning model. The split peaks are often associated with different TF motifs (Fig 2B). (PDF) [file pcbi.1004271.s005.pdf]

DNase-seq peaks  
(3717)

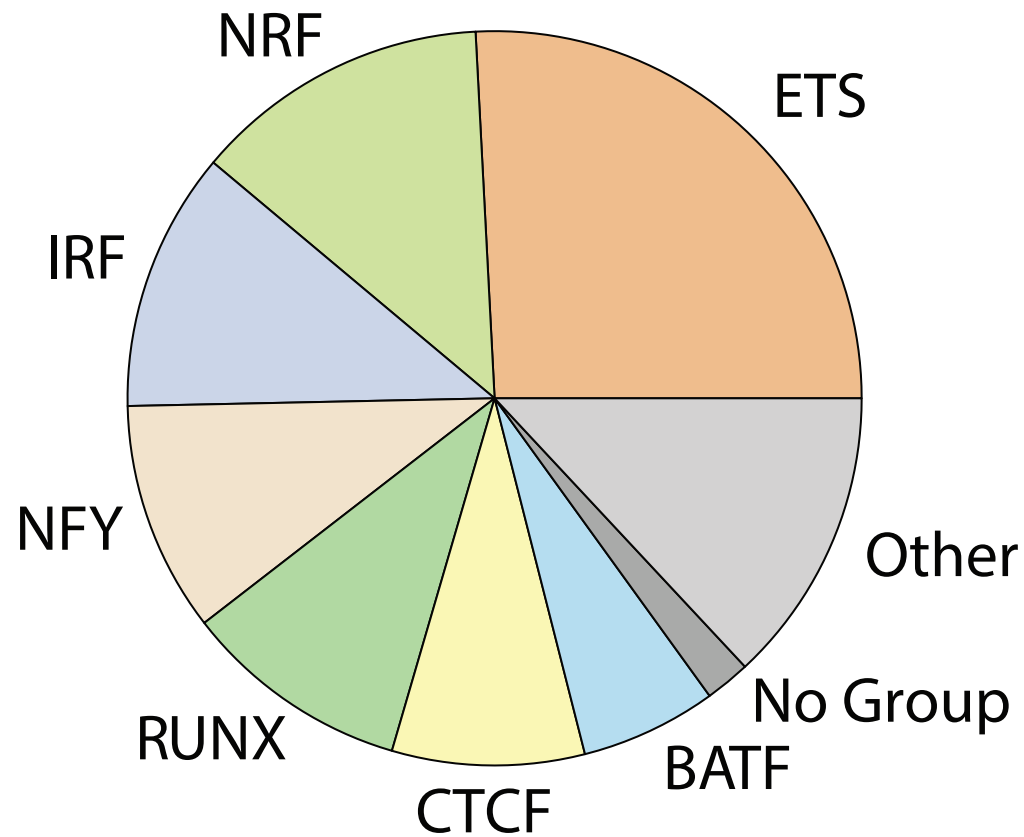

ATAC-seq peaks  
(3717)

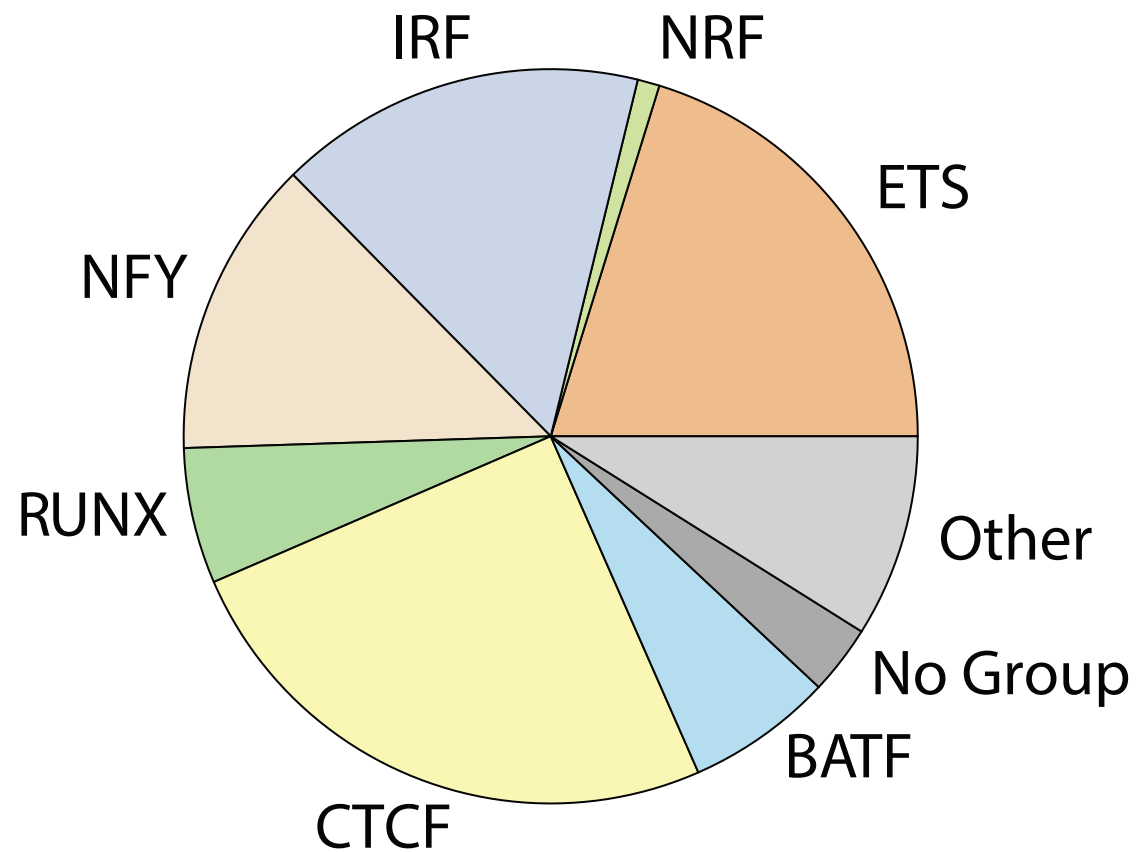

Supplement: S7 Fig — The enrichment of NRF and CTCF motifs in DNase-seq and ATAC-seq respectively is also observed in peaks common to both datasets. We identified common peaks by finding peaks that overlap by at least 90% after defining windows of 150 bases around the peak summits. (PDF) [file pcbi.1004271.s007.pdf]

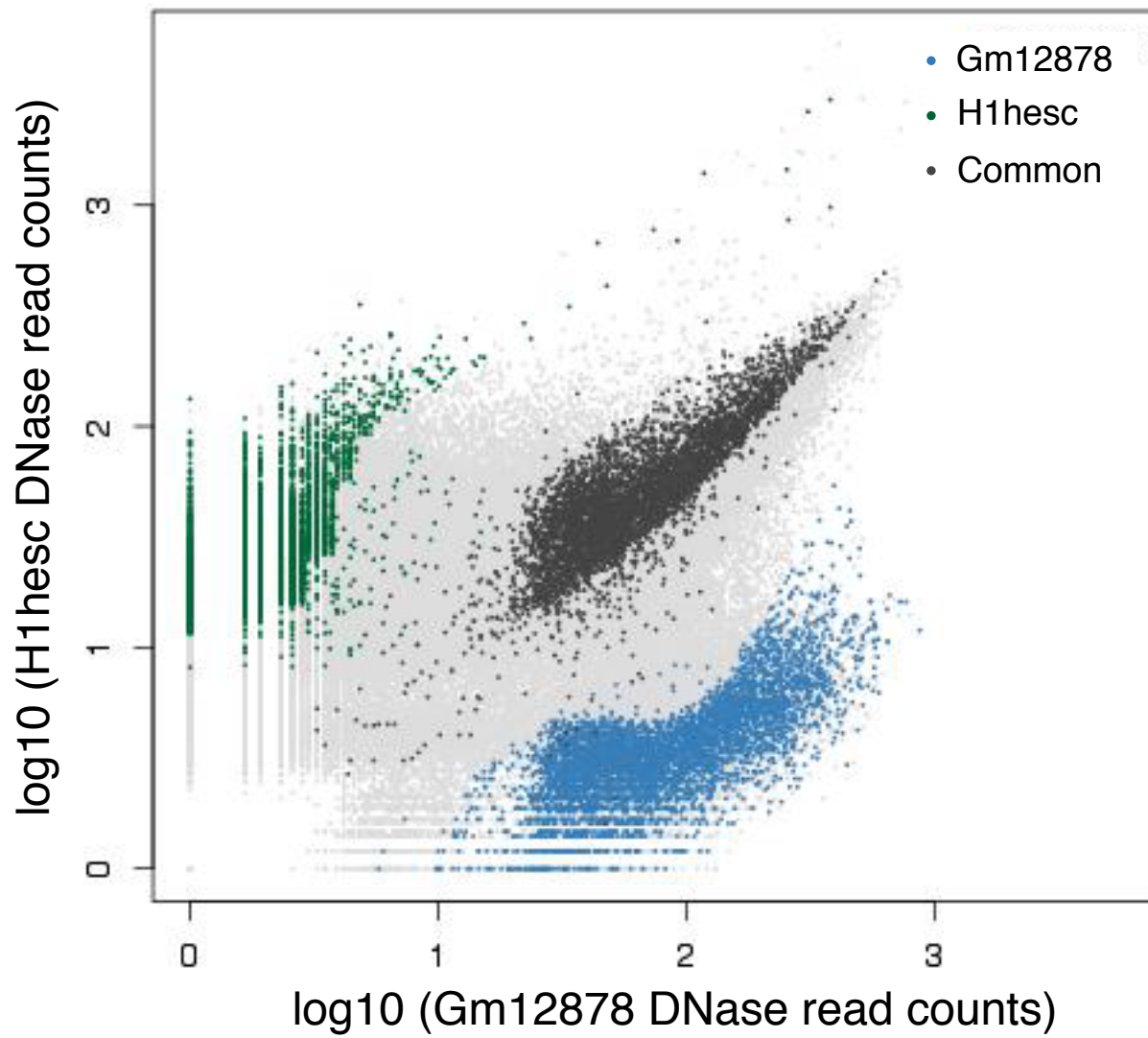

Supplement: S8 Fig — The scatterplot of log read counts of DNase peaks between two cell types shows both a large number of cell-type specific peaks as well as common peaks. DESeq [49] was used to identify the cell-type specific and common peaks. FDR corrected p-value of 0.01 was used for cell-type specific peaks whereas peaks with FDR corrected p-value > 0.25 were used as common peaks. The peaks in green and blue are used as H1-hESC and GM12878 specific peaks and peaks in black are used as common peaks for identification of motifs in Fig 2C. (PDF) [file pcbi.1004271.s008.pdf]

H1hesc DNase log read counts (replicate 2)

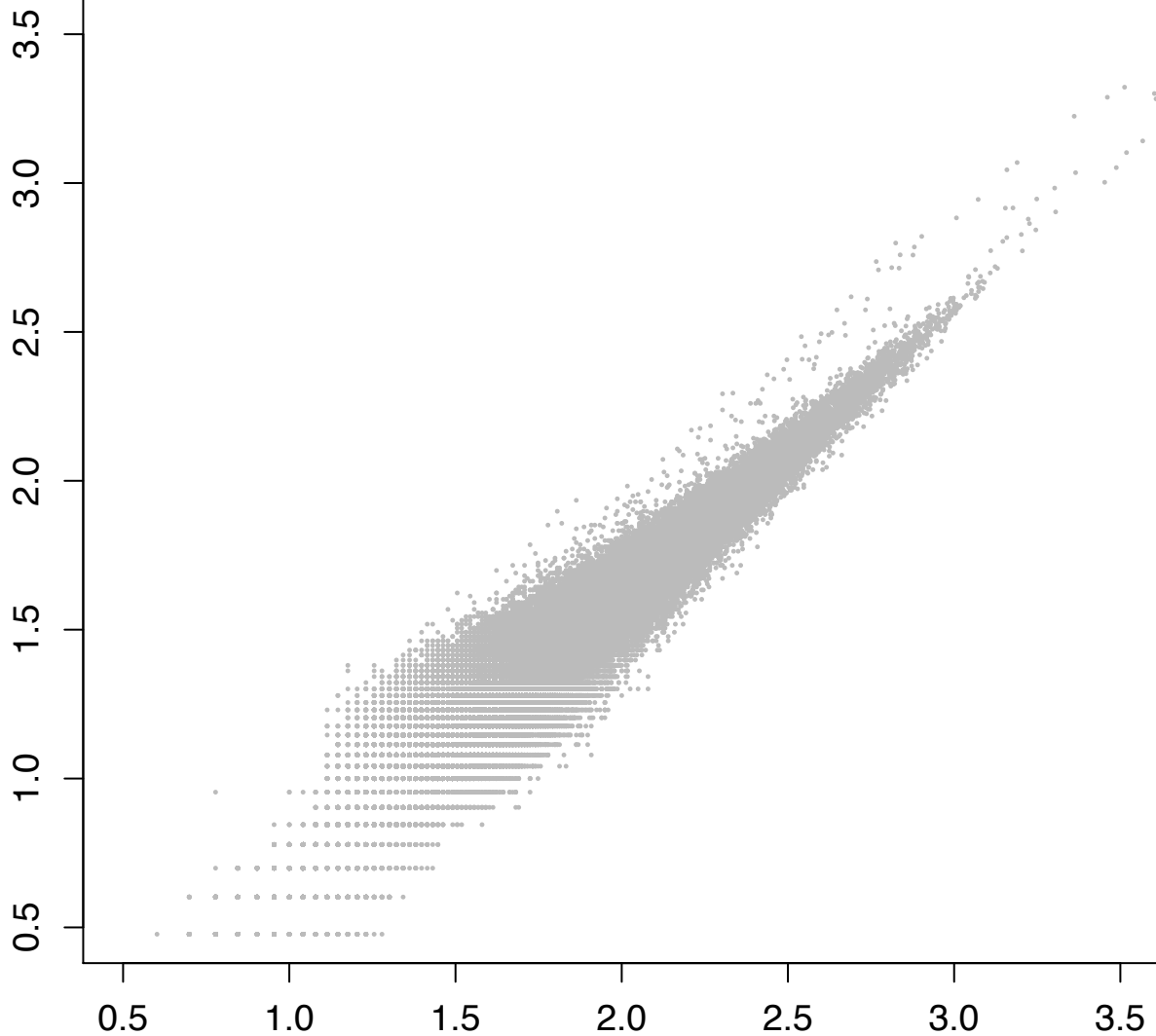

H1hesc DNase log read counts (replicate 1)

Supplement: S9 Fig — We use IDR with a cutoff of 0.01 to identify reproducible subpeaks in each cell type. The plot shows the identified subpeaks in H1-hESC. (PDF) [file pcbi.1004271.s009.pdf]

# Group 115 (positive class)

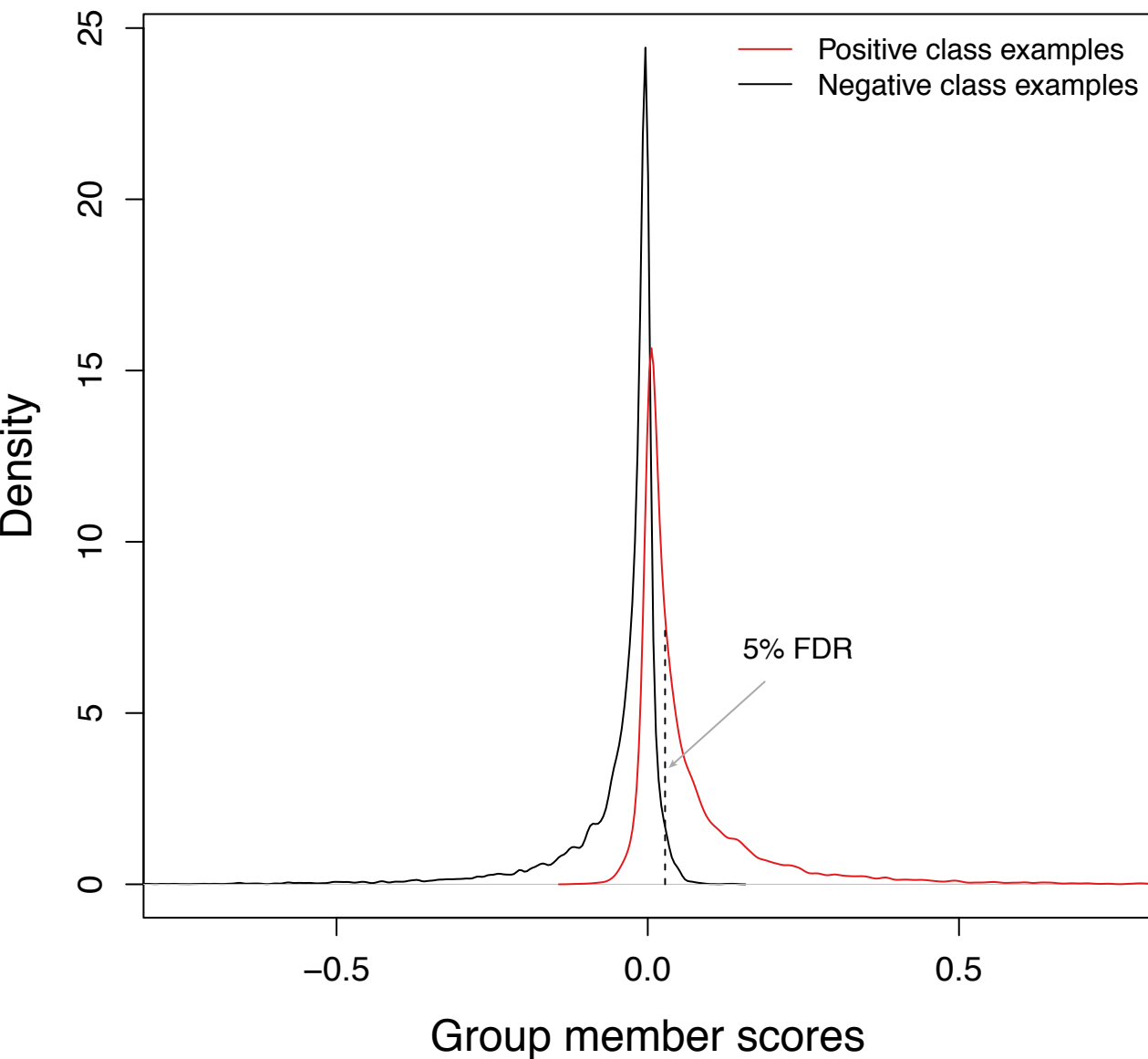

Supplement: S10 Fig — Group members for the positive class are determined using the scores of the negative class as the empirical null distribution (black line). We calculate an FDR-corrected p-value for each positive peak using this empirical null. A 5% FDR threshold was used in the association of peaks with groups. (PDF) [file pcbi.1004271.s010.pdf]

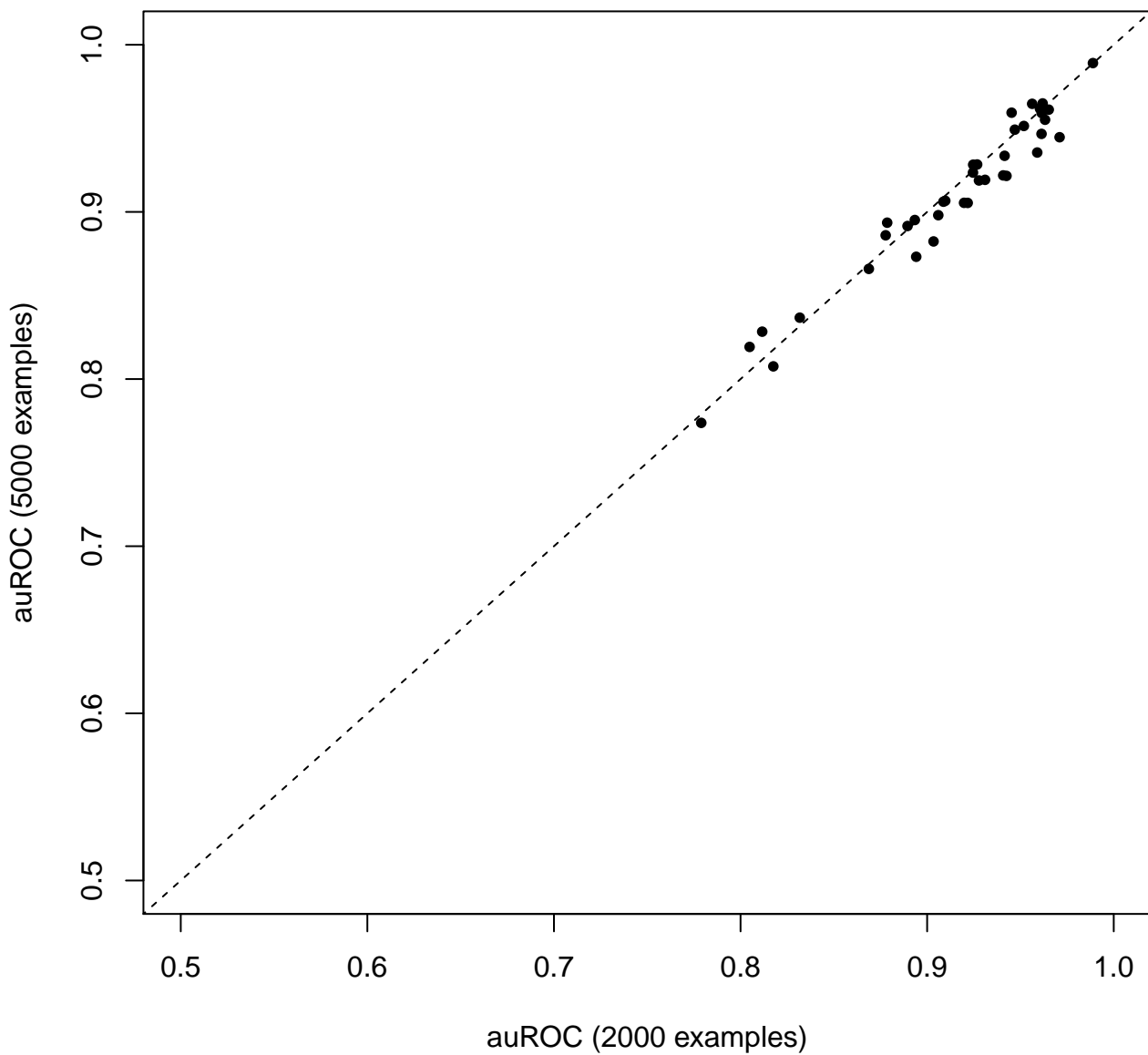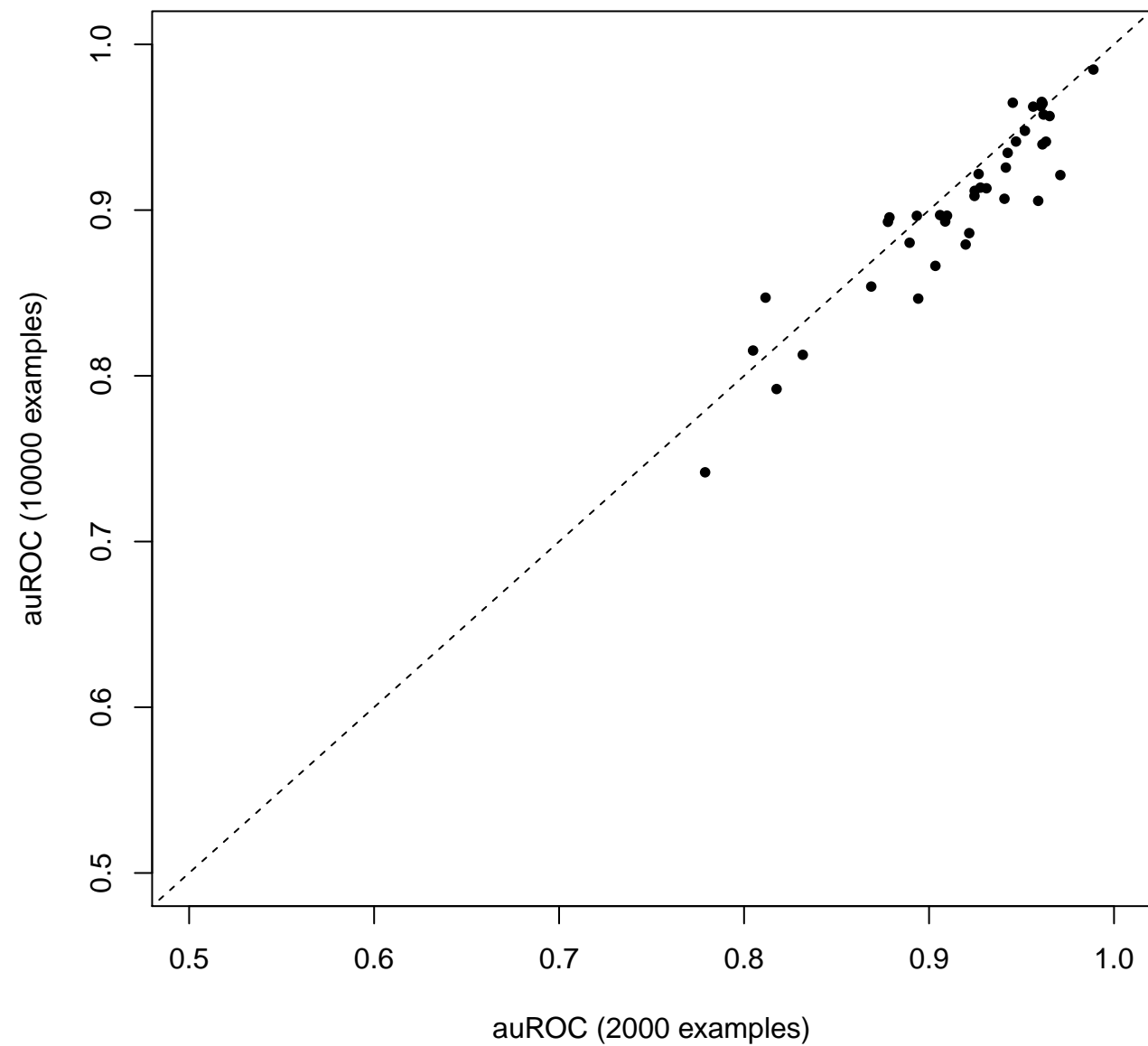

Supplement: S11 Fig — In order to test the effect of increasing noise in the training set, we used (a) 5000 examples and (b) 10000 examples for training and test in ChIP-seq experiments where sufficient peaks are available. Increasing the number of training examples does not significantly affect performance either with 5000 examples (p < 0.6, Wilcoxon rank sum test) or 10000 examples (p < 0.25, Wilcoxon rank sum test) (PDF) [file pcbi.1004271.s011.pdf]

A

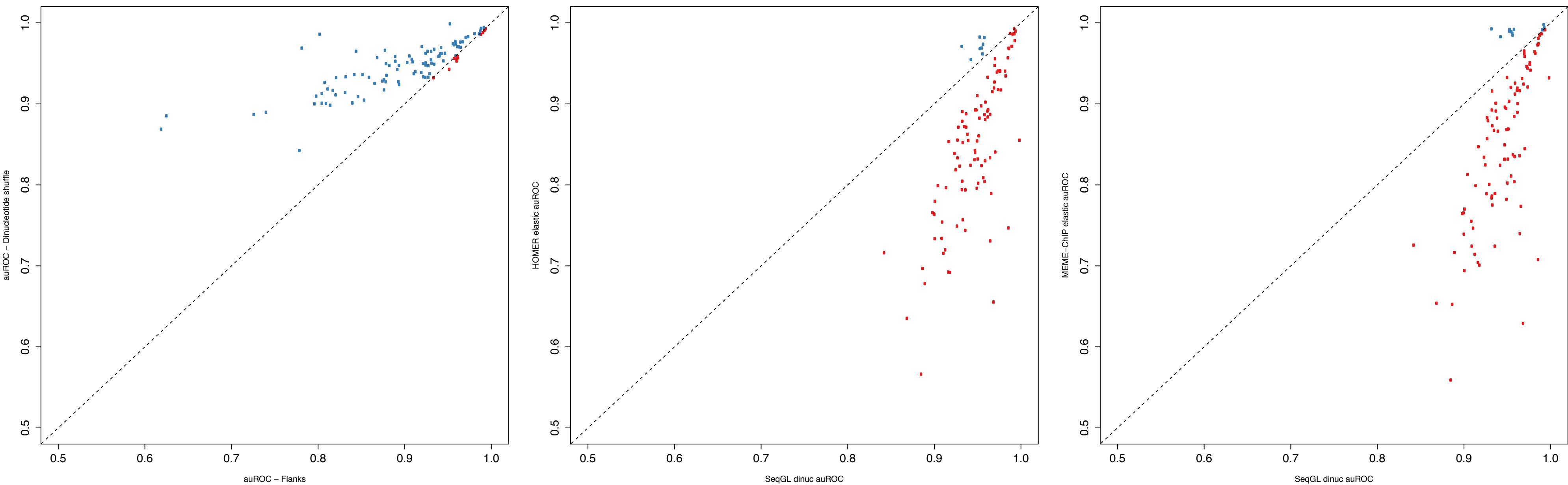

B

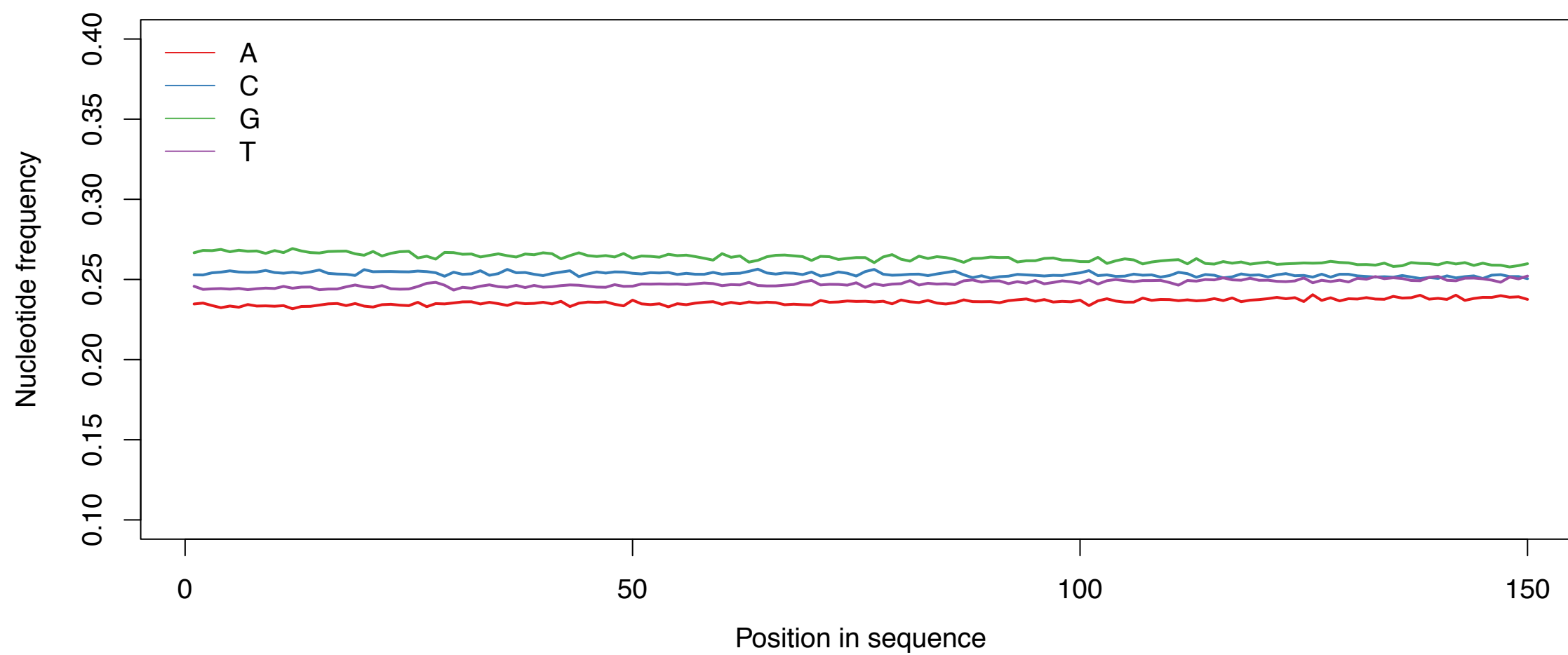

Supplement: S12 Fig — (A) We used dinucleotide shuffled sequences instead of flanking sequences as negatives and observe a significantly better SeqGL performance with shuffled sequences (p < 2e-7, Wilcoxon rank sum test). Thus shuffled sequences are “easy” negatives and do not present a strong adversary. Moreover, SeqGL using dinucleotide shuffled sequences as negatives significantly outperforms HOMER and MEME-ChIP on this task (p < 2e-10, Wilcoxon rank sum test). (B) Plot showing distribution of nucleotide frequencies in the negative flank sequences across all 105 ChIP-seq experiments. This shows that negative flank sequences are not enriched for polyA sequences. We also enumerated the number of low complexity sequences in the dataset (a sequence was defined to be low complexity if a particular nucleotide is repeated in 50% of sequence positions). <2% of the flank sequences were identified as low complexity (as opposed to <1% of peak sequences) indicating that flank sequences are not enriched for low complexity sequences. (PDF) [file pcbi.1004271.s012.pdf]
