# Supplementary material for: SeqGL Identifies Context-Dependent Binding Signals in Genome-Wide Regulatory Element Maps
Source: PLoS Comput Biol. 2015 May 27;11(5):e1004271. doi: 10.1371/journal.pcbi.1004271 (PMC4446265; doi:10.1371/journal.pcbi.1004271)
Supplement: S6 Fig — A number of groups in GM12878 DNase-seq analysis are associated with motifs that only partially match to known motifs. These motifs are potentially novel motifs that have not been characterized or variants of known motifs. (PDF) [file pcbi.1004271.s006.pdf]

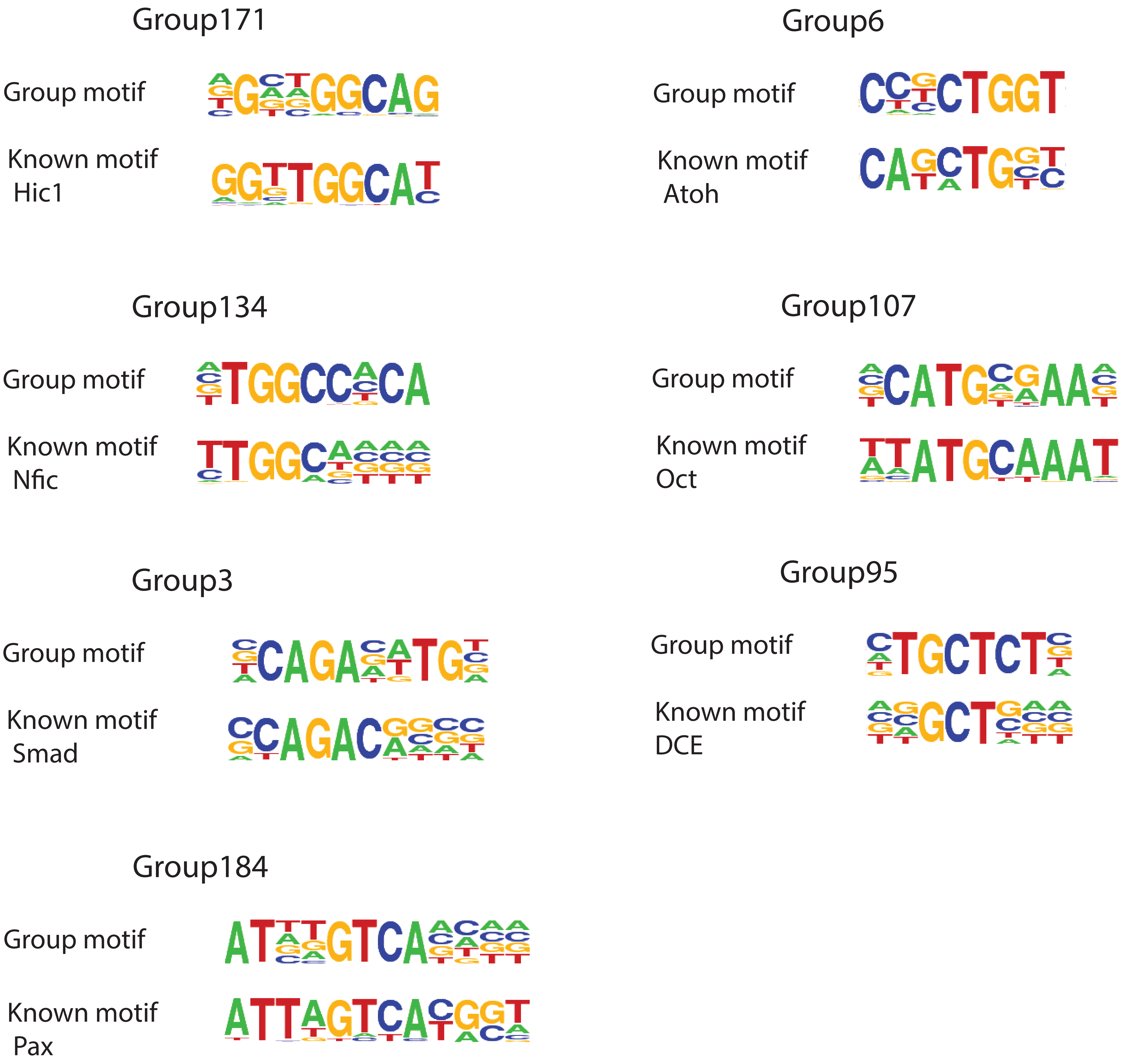
Group171
Group motif
Known motif
Hic1
Group6
Group motif
Known motif
Atoh
Group134
Group motif
Known motif
Nfic
Group107
Group motif
Known motif
Oct
Group3
Group motif
Known motif
Smad
Group95
Group motif
Known motif
DCE
Group184
Group motif
Known motif
Pax
